# Factors associated with long-term use of digital devices in the electronic Framingham Heart Study

**DOI:** 10.1038/s41746-022-00735-1

**Published:** 2022-12-27

**Authors:** Chathurangi H. Pathiravasan, Yuankai Zhang, Xuzhi Wang, Ludovic Trinquart, Emelia J. Benjamin, Belinda Borrelli, David D. McManus, Vik Kheterpal, Honghuang Lin, Nicole L. Spartano, Eric Schramm, Chunyu Liu, Joanne M. Murabito

**Affiliations:** 1 Department of Biostatistics, Boston University School of Public Health, Boston, MA USA; 2 Institute for Clinical Research and Health Policy Studies, Tufts Medical Center, Boston, MA USA; 3 Tufts Clinical and Translational Science Institute, Tufts University, Boston, MA USA; 4 Boston University’s and National Heart, Lung, and Blood Institute’s Framingham Heart Study, Framingham, MA USA; 5 Section of Cardiovascular Medicine, Department of Medicine, Boston Medical Center, Boston University Chobanian & Avedisian School of Medicine and Department of Epidemiology, Boston University School of Public Health, Boston, MA USA; 6 Henry M. Goldman School of Dental Medicine, Center for Behavioral Science Research, Department of Health Policy & Health Services Research, Boston University, Boston, MA USA; 7 Department of Medicine, University of Massachusetts Chan Medical School, Worcester, MA USA; 8 Department of Quantitative Health Sciences, University of Chan Massachusetts Medical School, Worcester, MA USA; 9 CareEvolution, Ann Arbor, MI USA; 10 Section of Endocrinology, Diabetes, Nutrition, and Weight Management, Boston University Chobanian & Avedisian School of Medicine, Boston, MA USA; 11 Section of General Internal Medicine, Department of Medicine, Boston University Chobanian & Avedisian School of Medicine and Boston Medical Center, Boston, MA USA

**Keywords:** Epidemiology, Research data

## Abstract

Long-term use of digital devices is critical for successful clinical or research use, but digital health studies are challenged by a rapid drop-off in participation. A nested e-cohort (eFHS) is embedded in the Framingham Heart Study and uses three system components: a new smartphone app, a digital blood pressure (BP) cuff, and a smartwatch. This study aims to identify factors associated with the use of individual eFHS system components over 1-year. Among 1948 eFHS enrollees, we examine participants who returned surveys within 90 days (*n* = 1918), and those who chose to use the smartwatch (*n* = 1243) and BP cuff (*n* = 1115). For each component, we investigate the same set of candidate predictors for usage and use generalized linear mixed models to select predictors (*P* < 0.1, *P* value from *Z* test statistic), adjusting for age, sex, and time (app use: 3-month period, device use: weekly). A multivariable model with the predictors selected from initial testing is used to identify factors associated with use of components (*P* < 0.05, *P* value from *Z* test statistic) adjusting for age, sex, and time. In multivariable models, older age is associated with higher use of all system components. Female sex and higher education levels are associated with higher completion of app-based surveys whereas higher scores for depressive symptoms, and lower than excellent self-rated health are associated with lower use of the smartwatch over the 12-month follow-up. Our findings show that sociodemographic and health related factors are significantly associated with long-term use of digital devices. Future research is needed to test interventional strategies focusing on these factors to evaluate improvement in long-term engagement.

## Introduction

Smartphone apps and digital devices provide ways to change a range of health behaviors and engage individuals in disease self-management including encouraging physical activity, monitoring blood pressure (BP), and addressing behavioral determinants of cardiovascular diseases (CVDs)^1–3^. However, studies have shown rapid disengagement with mobile health devices, leading to inadequate maintenance of behavior change^4,5^. Most digital studies have evaluated adherence over a short time period^5^ with limited investigation of sustained device usage over 10–12 months^6^.

Identifying predictors of long-term use of digital devices may inform future guidelines and interventions to support user engagement. Since few studies have deployed integrated solutions of digital devices for CVD phenotyping among cohort study participants, little is known about factors related to long-term use or disuse of such systems. The Health eHeart Study is an e-Cohort that used smartphones, and Bluetooth BP cuff technology to study heart disease^7^. In this cardiovascular study, 28.6% of consented participants completed survey data and a subset of 251 participants who owned devices sent device based BP data among 42,828 registered participants. In the Asthma Mobile Health Study, 6470 participants (85%) responded to at least one survey at baseline among 7593 enrolled participants. However only 175 users completed the 6-month milestone surveys^8^. Michigan Predictive Activity & Clinical Trajectories in Health (MIPACT) is a large (*n* = 6765) observational study that uses a smartphone application, a smartwatch, and a wireless BP cuff to collect CVD risk factor data^9^. The study described key wearable signals across age, sex, race, ethnicity, and clinical phenotypes. It also investigated two user groups based on the level of task completion: basic completion and comprehensive completion and the study had lower attrition compared to previous studies. During the 45-day collection phase, 98.3% completed the basic completion requirement while 86.3% provided comprehensive data.

Long-term adherence is one of the fundamental problems in digital health studies^10^. Median participant retention in the MyHeart Counts Cardiovascular Health Study and Asthma Study varies from 9 days and 12 days respectively^8,11^. Thus, there is a need to investigate long-term use of digital technologies and its predictive factors. Studies investigating long-term use of mobile apps or digital devices have focused on individuals with chronic medical conditions^12–14^, had low sample sizes^13,15^, or studied a limited number of factors for association with device use^16^. Data for sustained device use and related factors were collected from self-reported questionnaires, and such data may be subject to social desirability bias^17^.

In 2016, the Framingham Heart Study (FHS) introduced a nested e-cohort (eFHS) to evaluate CVD phenotypes using digital devices. The eFHS contains three system components: a smartphone app and two digital devices (a smartwatch and a digital BP cuff)^18^. Smartphone app-based surveys are deployed at enrollment and every 3 months (Supplementary Table 1). Participants’ steps and heart rates are collected daily from the smartwatch while BP measurements are collected weekly from the digital blood pressure cuff (BP cuff). At each FHS in-person research exam, sociodemographic, health behaviors, and health variables are collected, providing the opportunity to evaluate contributing factors for sustained use of digital devices. We aim to examine temporal trends in the use of the three individual eFHS system components over 52 weeks and to identify factors associated with long-term use of each component.

## Results

### Characteristics and trends of digital device use

**Fig. 1:**
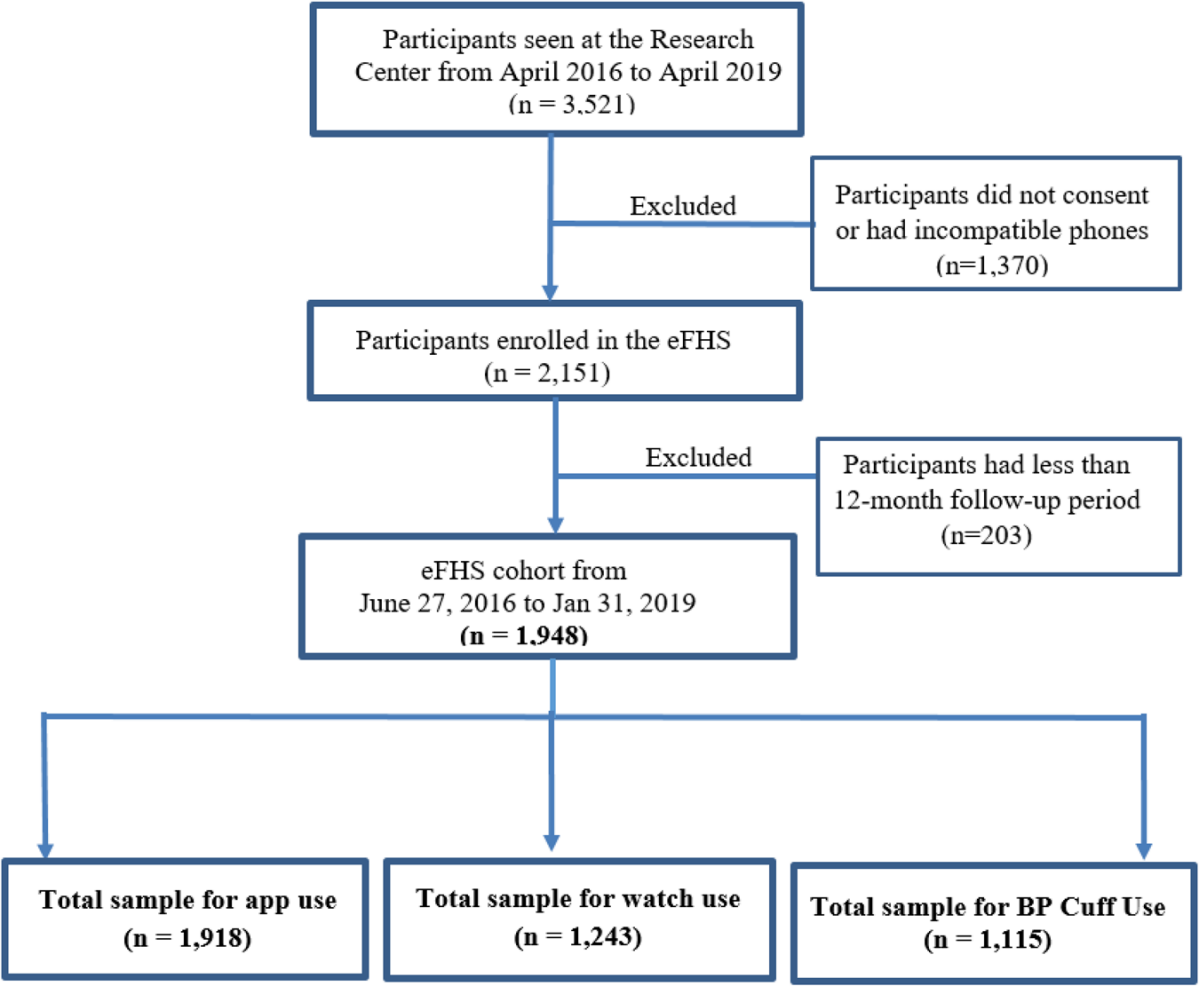
Sample Selection for Analysis of eFHS System Components. In the eFHS cohort (*n* = 1948), 30 participants took more than 90 days to return baseline surveys and were excluded as it was difficult to distinguish baseline and follow-up surveys. The total sample for app use (*n* = 1918) includes participants who didn’t return surveys (*n* = 213) and who returned surveys (*n* = 1705). The sample for watch use (*n* = 1243) consists of participants who sent data from the watch (*n* = 1185) and who took the watch but never sent any data (*n* = 58). The total sample of BP cuff use includes participants who returned BP (*n* = 969) data and who took the BP cuff but never sent any BP data (*n* = 146).

Among 1918 eFHS enrollees who used the app, 1705 individuals completed at least one baseline smartphone app survey within 90 days, 1243 participants chose to use a smartwatch and 1125 participants chose to use a wireless BP device (Fig. 1). Among participants who used digital devices, 969 participants returned BP measurements, and 1185 participants transmitted smartwatch steps or heart rate data. Among these participants, 798 individuals used all three system components (Supplementary Fig. 1). Characteristics of samples with app use and digital device use were similar (Table 1). The majority of eFHS participants were middle aged (mean age 53 years), White (>90%) and had high educational levels (Table 1). We also compared the characteristics between those who enrolled in eFHS and those who did not. Those who enrolled in eFHS had a lower prevalence of hypertension (26% vs. 37%), and CVD (3.0 % vs. 5.4%) compared to participants who did not enroll in eFHS (Supplementary Table 2). We further investigated characteristics of our eFHS study sample. eFHS participants were also more likely to be iPhone users (iPhone: 85.8%, Android: 14.2%) and iPhone users more likely to complete baseline surveys (iPhone users: 92%, Android users: 71%). Android users were not eligible to receive the digital devices (either smartwatch or BP cuff) because the Android OS was not compatible with the devices. iPhone users who took the digital devices were more likely to complete baseline surveys than iPhone users who did not take digital devices (digital device users: 94%, not a digital device user 79%).

**Table 1:** Characteristics of participants in eFHS: Smartphone App Use, Smartwatch Use, and Digital BP Cuff Use.

Variable^b^	Smartphone App^a^ (*n* = 1918)	Smartwatch^a^ (*n* = 1243)	BP Cuff^a^ (*n* = 1115)
Age, years, mean (SD)	52.8 (8.7)	52.6 (8.7)	53.0 (8.6)
Age Groups, *n* (%)			
* Age* < 45	328 (17.1)	223 (17.9)	178 (16.0)
45 ≤ *Age* < 55	772 (40.3)	505 (40.6)	451 (40.4)
55 ≤ *Age* < 65	655 (34.2)	415 (33.4)	398 (35.3)
* Age* ≥ 65	163 (8.5)	100 (8.0)	92 (8.3)
Female sex, *n* (%)	1093 (57.0)	735 (59.1)	655 (58.7)
Race (White), *n* (%)	1783 (93.0)	1140 (91.7)	1029 (92.3)
Body mass index, kg/m^2c^, mean (SD)	28.2 (5.6)	28.4 (5.7)	27.7 (4.9)
Systolic blood pressure, mmHg^d^, mean (SD)	119 (14)	118 (14)	119 (14)
Diastolic blood pressure, mmHg^e^, mean (SD)	76 (8)	76 (8)	76 (8)
Current smoking, *n* (%)^f^	112 (5.8)	67 (5.4)	55 (4.9)
Diabetes mellitus, *n* (%)^g^	120 (6.3)	71 (5.7)	61 (5.5)
Hypertension, *n* (%)	498 (26.0)	324 (26.1)	294 (26.4)
Prevalent cardiovascular disease, *n* (%)	59 (3.1)	37 (3.0)	36 (3.2)
Physical activity index^h^, mean (SD)	32.5 (5.1)	33.3 (4.9)	33.6 (5.1)
Education Level Achieved^i^			
Less than or completed high school	175 (9.1)	102 (8.2)	98 (8.8)
Completed some college	462 (24.1)	288 (23.2)	254 (22.8)
Bachelor’s degree	733 (38.2)	483 (38.9)	428 (38.4)
Graduate or professional degree	548 (28.6)	370 (29.8)	335 (30.0)
Married, living as married, living with partner, *n* (%)^j^	1436 (74.9)	939 (75.5)	854 (76.6)
Subjective Health, *n* (%)^k^			
Excellent	461 (24.0)	299 (24.1)	294 (26.3)
Very Good	931 (48.5)	591 (47.5)	525 (47.1)
Good	448 (23.4)	301 (24.2)	258 (23.1)
Poor/Fair	78 (4.1)	52 (4.2)	38 (3.4)
Depressive Symptoms Scores, mean (SD)	6 (7)	6 (7)	6 (7)
Employed Full Time, *n* (%)^l^	1347 (70.2)	879 (70.7)	797 (71.5)
Cholesterol level, mg/dL^m^, mean (SD)	190.5 (35.7)	190.6 (36.3)	190.8 (36.4)
Current Alcohol Consumption, *n* (%)^n^	1561 (81.4)	1012 (81.4)	905 (81.2)

^a^Data reflect enrollment up to January 28, 2019.

^b^Characteristics were presented as mean ± standard deviation (SD) for continuous variables, numbers and percentages for nominal variables.

^c^Missing data: App *n* = 1, Smartwatch *n* = 1, BP Cuff *n* = 1 (If data was missing for a variable at exam 3, we used data from a prior attended exam).

^d^Missing data: App *n* = 2, Smartwatch *n* = 1, BP Cuff *n* = 1 (If data was missing for a variable at exam 3, we used data from a prior attended exam).

^e^Missing data: App *n* = 3, Smartwatch *n* = 2, BP Cuff *n* = 2 (If data was missing for a variable at exam 3, we used data from a prior attended exam).

^f^Missing data: App *n* = 1, Smartwatch *n* = 1, BP Cuff *n* = 1 (If data was missing for a variable at exam 3, we used data from a prior attended exam).

^g^Missing data: App *n* = 7, Smartwatch *n* = 4, BP Cuff *n* = 3 (If data was missing for a variable at exam 3, we used data from a prior attended exam).

^h^Missing data: App *n* = 1, Smartwatch *n* = 0, BP Cuff *n* = 1 (If data was missing for a variable at exam 3, we used data from a prior attended exam).

^i^Missing data: App *n* = 9, Smartwatch *n* = 5, BP Cuff *n* = 6 (If data was missing for a variable at exam 3, we used data from a prior attended exam).

^j^Missing data: App *n* = 13, Smartwatch *n* = 7, BP Cuff *n* = 8 (If data was missing for a variable at exam 3, we used data from a prior attended exam).

^k^Missing data: App *n* = 1, Smartwatch *n* = 1, BP Cuff *n* = 0 (If data was missing for a variable at exam 3, we used data from a prior attended exam).

^l^Missing data: App *n* = 9, Smartwatch *n* = 8, BP Cuff *n* = 6 (If data was missing for a variable at exam 3, we used data from a prior attended exam).

^m^Missing data: App *n* = 6, Smartwatch *n* = 4, BP Cuff *n* = 3 (If data was missing for a variable at exam 3, we used data from a prior attended exam).

^n^Missing data: App *n* = 5, Smartwatch *n* = 3, BP Cuff *n* = 3 (If data was missing for a variable at exam 3, we used data from a prior attended exam).

**Fig. 2:**
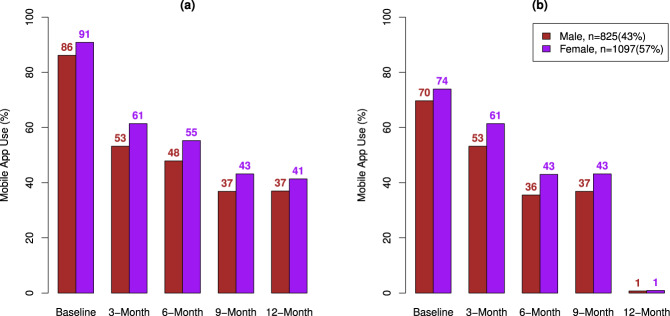
Smartphone App Use by sex. **a** Proportions of participants who complete at least one survey at a given 3-month period stratified by sex. **b** Proportions of participants who complete all surveys at a given 3-month period stratified by sex.

**Fig. 3:**
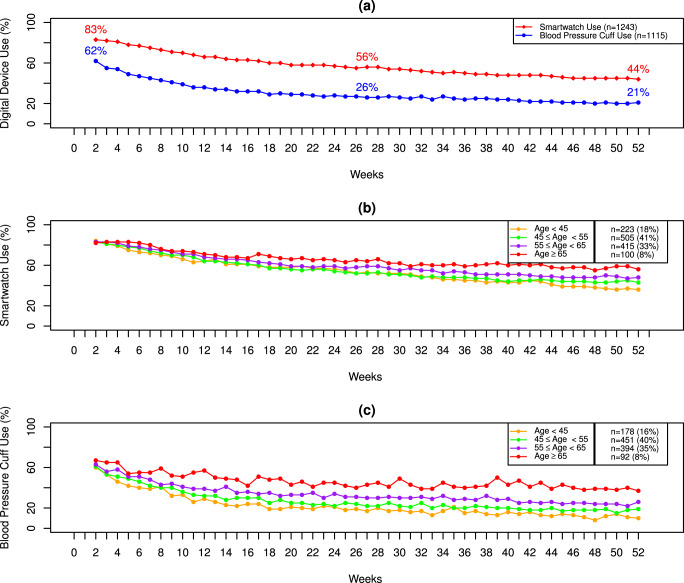
Trends of Smartwatch and Digital BP Cuff Use over 52 weeks. **a** Digital device use over 52 weeks. **b** Smartwatch Use Stratified by Age. **c** Digital Blood Pressure Cuff Use stratified by Age.

Trends of digital device use were considered based on observed values. The survey return rate decreased over time (Fig. 2). Both female and male participants had high survey return rates at baseline. Women were more likely to complete surveys at each 3-month interval compared to men (Fig. 2). Smartwatch use and BP cuff use also decreased over 52 weeks (Fig. 3a). eFHS participants were more likely to use the smartwatch than the BP cuff over 52 weeks (Smartwatch use: 56% and BP Cuff Use: 26% at 26 weeks, Smartwatch use: 44% and BP Cuff Use: 21% at 52 weeks). Older participants were more likely to use these digital devices than younger adults (Fig. 3b and c).

### Factors associated with smartphone app use

We defined smartphone survey app usage in two ways (Supplemental Table 3). First, smartphone app use was defined based on whether at least one survey was completed in a given survey wave. For each candidate predictor (Supplementary Fig. 2), generalized linear mixed models (GLMMs) were fitted (Supplementary Fig. 3) and predictors with *P* < 0.1 (*P* values derived using *Z* test statistic from GLMMs) from individual model testing (Supplementary Table 4) were included in the final multivariable model adjusting for age, sex, and 3-month period. In the multivariable model, participants in the older age group (≥65 years) and middle age group (55–65 years) had higher survey app usage compared to younger participants (<45 years) over the 12-month period (Table 2). Women had higher survey adherence (OR 2.69, 95% Confidence Interval (CI) [1.68–4.31], *P* < 0.001 where *P* values derived using *Z* test statistic from GLMMs) compared to men. College graduates or participants with a professional degree were more likely to use the survey app compared to participants who completed high school or less than high school (OR 2.51, 95% CI [1.04–6.10], *P* = 0.041 where *P* values derived using *Z* test statistic from GLMMs). Next, smartphone app use was defined based on whether all surveys were completed in a given survey wave (3-month period) and significant predictors in individual model testing (Supplementary Table 5) were included in the final multivariable model. Based on the multivariable model, women and participants who completed bachelor’s degree, graduate or professional degree were more likely to complete all surveys at a given 3-month period (Table 2).

**Table 2:** Factors associated with smartphone app use: Results of multivariable models.

Predictors	Model for smartphone app use Completing at least one survey at a given 3-month interval, *N* = 9590, *n* (eFHSID) = 1918		Model for smartphone app use Completing all surveys at a given 3-month interval, *N* = 9590, *n* (eFHSID) = 1918	
	OR (95% CI)	*P* value^a^	OR (95% CI)	*P* value^a^
Age				
* Age* < 45	–	–	–	–
45 ≤ *Age* < 55	1.18 (0.62–2.27)	0.610	0.96 (0.65–1.40)	0.817
55 ≤ *Age* < 65	2.59 (1.31–5.12)	0.006	1.32 (0.89–1.96)	0.174
* Age* ≥ 65	6.42 (2.36–17.50)	<0.001	1.54 (0.88–2.71)	0.132
Sex (Female)	2.69 (1.68–4.31)	<0.001	1.77 (1.35–2.32)	<0.001
Subjective Health				
Health Excellent	–	–	–	–
Very Good	0.95 (0.53–1.70)	0.868	0.95 (0.68–1.33)	0.757
Good	0.68 (0.34–1.35)	0.267	0.81 (0.54–1.21)	0.306
Poor/Fair	0.99 (0.28–3.56)	0.996	0.79 (0.38–1.66)	0.538
Depressive Symptoms Scores (Rescale)	0.86 (0.68–1.09)	0.205	0.87 (0.75–1.00)	0.047
Current Smoking	0.43 (0.16–1.14)	0.089	0.82 (0.46–1.46)	0.491
Education Level				
Less than or completed high school	–	–	–	–
Completed some college	1.50 (0.62–3.64)	0.365	1.62 (0.96–2.72)	0.072
Bachelor’s degree	2.11 (0.90–4.97)	0.087	1.89 (1.14–3.14)	0.013
Graduate or professional degree	2.51 (1.04–6.10)	0.041	2.19 (1.30–3.69)	0.003
Married	–	–	1.26 (0.92–1.72)	0.152

The reference groups, respectively, were age < 45; Male sex; Health Excellent; not currently smoking, Education less than or completed high school; not married.

*N* = observations; *n* = unique individuals; µ = mean, *σ* = standard deviation; predictor = x; Rescale (x) = $\frac{{x - \mu }}{\sigma }$

*N* = 7590, *n* = 1918 (For survey analysis).

Model: Completing at least one survey at a given 3-month interval (Covariates: age, sex, subjective health, depressive symptoms, education level, and current smoking status).

Model: Completing all surveys at a given 3-month interval (Covariates: age, sex, subjective health, depressive symptoms, education level, and marital status).

Time of use (3-month period)) was adjusted in both models (not shown in the table).

Depressive Symptoms Scores (Rescale): µ = 6.05, *σ* = 6.70.

^a^*P* values are derived using *Z* test statistic from generalized linear mixed models (GLMM).

In secondary analyses (Supplementary Fig. 4), we investigated whether the effect of age and sex in the multivariable model differs according to time of use based on whether at least one survey was completed at each time period. In the interaction models, age and time of use interaction terms were statistically significant (*P* value interaction term Age × 9-month < 0.001 and *P* value for the interaction term Age × 12-month < 0.001, Here *P* values derived using *Z* test statistic from GLMMs) while sex and time of use interaction terms were not significant (Supplementary Table 6). In the stratified analysis with respect to each 3-month period, we observed that statistical significance (effect estimates and *P* values of age) varied across each 3-month interval (Supplementary Table 6) but the directionality of the relationship between age and app use was similar to the non-stratified analysis. Next, we performed an interaction analysis with respect to age groups (age ≤53 and age >53, where median age = 53) and each predictor (Supplementary Table 7). All interaction terms were not statistically significant (*P* values for Age groups × each predictor >0.05, *P* values derived using *Z* test statistic from GLMMs).

### Factors associated with use of smartwatch and wireless blood pressure cuff

We defined weekly smartwatch usage as yes = watch wear for ≥1 day for ≥5 h per day, vs. no and weekly BP cuff usage as yes = BP measurement returns ≥1 per week, vs. no (Supplemental Table 3). Significant predictors from individual GLMMs testing (Supplementary Table 8) were included in the final multivariable model for smartwatch use adjusting for age, sex, and weeks of watch use. In the final multivariable model for watch use, age was a significant predictor. Participants aged ≥ 65 and age 55–65 years were more likely to use the watch compared to participants with age <45 years (Table 3). Participants who reported good health had lower watch use (OR 0.36, 95% CI [0.16–0.79], *P* = 0.001 where *P* values derived using *Z* test statistic from GLMMs) compared to participants who reported excellent health. Individuals who reported health as poor or fair had lower smartwatch use compared to participants who reported excellent health (OR 0.68, 95% CI [0.16–2.85], *P* = 0.594 where *P* derived using *Z* test statistic from GLMMs). However, the result was not statistically significant. There were only 4.2% of participants with poor/fair health, and therefore, we may have been underpowered to detect an association. Higher depressive symptoms scores (increase of one standard deviation away from the mean of depressive symptoms scores) were associated with lower odds of watch use (OR 0.76, 95% CI [0.59–0.98], *P* = 0.038 where *P* values derived using *Z* test statistic from GLMMs). Similarly, significant predictors from initial testing (Supplementary Table 9) were included in the final multivariable model for BP cuff use. Age (age group ≥ 65 vs. age <45: OR = 5.83; 95% CI, 3.22–10.53 & age group [55,65) vs. age <45: OR = 2.58; 95% CI, 1.70–3.93) remained significant in the multivariable model (Table 3).

**Table 3:** Factors associated with digital device use: results of multivariable models.

Predictors	Model for Smartwatch Use, *N* = 64636, *n* (eFHSID) = 1243		Model for Digital Blood Pressure Cuff Use, *N* = 57980, *n* (eFHSID) = 1115	
	OR (95% CI)	*P* value^a^	OR (95% CI)	*P* value^a^
Age Groups				
* Age* < 45	–	–	–	–
45 ≤ *Age* < 55	1.30 (0.64–2.65)	0.463	1.53 (1.02–2.31)	0.041
55 ≤ *Age* < 65	2.13 (1.02–4.44)	0.045	2.59 (1.70–3.93)	<0.001
Age ≥ 65	3.72 (1.27–10.88)	0.016	5.82 (3.22–10.53)	<0.001
Sex (Female)	1.58 (0.94–2.66)	0.085	1.07 (0.81–1.42)	0.642
Subjective Health				
Health Excellent	–	–	–	–
Very Good	0.75 (0.39–1.44)	0.387	0.92 (0.65–1.29)	0.616
Good	0.36 (0.16–0.79)	0.001	0.73 (0.49–1.10)	0.130
Poor/Fair	0.68 (0.16–2.85)	0.594	1.18 (0.52–2.71)	0.693
Depressive Symptoms Scores (Rescale)	0.76 (0.59–0.98)	0.038	0.90 (0.77–1.04)	0.141
BMI (Rescale)	0.87 (0.66–1.15)	0.336	–	–
Current Smoking	0.41 (0.14–1.25)	0.117	–	–

The reference groups, respectively, were age < 45, Male sex; Health Excellent; not currently smoking.

*N* = observations; *n* = unique individuals; µ = mean, *σ* = standard deviation; predictor = x; Rescale (x) = $\frac{{x - \mu }}{\sigma }$

*N* = 64636, *n* = 1243 (Smartwatch Analysis); *N* = 57980, *n* = 1115 (BP Cuff Analysis).

Weeks (Rescale) was adjusted in the model (not shown in the table): µ = 26.5, *σ* = 15 (Smartwatch); µ = 26.5, *σ* = 15 (Digital BP Cuff).

Depressive Symptoms Scores (Rescale): µ = 5.99, *σ* = 6.72 (Smartwatch Analysis); µ = 5.85, *σ* = 6.80 (Digital BP Cuff Analysis).

BMI (Rescale): µ = 28.42, σ = 5.69 (Smartwatch Analysis).

^a^*P* values are derived using *Z* test statistic from generalized linear mixed models (GLMM).

To investigate effect modification of time of use and age, interaction models were considered (Supplementary Fig. 4) and stratified analyses were performed for time of use (weeks >26 and weeks ≤26) and different age groups (age ≤53 and age >53). In the interaction models, there was a significant interaction between sex and watch use time periods (Weeks ≤26 and Weeks >26) as the interaction *P* values were <0.05 where *P* values derived using *Z* test statistic from GLMMs (Supplementary Table 10). We also observed a significant interaction between age and watch use time periods (Supplementary Table 10). Results for the BP Cuff subgroup analysis were similar. Age had a significant interaction with BP Cuff Use periods, but sex did not (Supplementary Table 11). To investigate whether age was an effect modifier, we tested interaction term of age groups and each characteristic (Supplementary Tables 12–13). However, none of the interaction terms in the GLMMs were significant at *P* < 0.05 (*P* value derived using *Z* test statistic from GLMMs). The relationship between watch use and BP cuff use was investigated with the subgroup of participants who used both devices. There was a strong correlation between watch use and BP cuff use (Coefficients of determinant *R*^*2*^ = 0.9, *n* = 937, Supplementary Fig. 5).

## Discussion

Digital health technologies provide opportunities to transform the delivery of clinical care and optimize clinical research. The transformation of healthcare has been accelerated during the COVID pandemic as patients increasingly wish to utilize remote or telemedicine tools^19^. To realize the benefits of digital technologies, strategies to address the challenges with user attrition and long-term engagement need to be developed. This study identified factors associated with engagement over a 1-year period of three digital device system components for CVD monitoring in the eFHS, a modestly sized (*n* ≈ 1948) community-based sample of middle-aged adults unselected for any health condition. eFHS system components had higher adherence (>80% for smartphone app survey return) at baseline that declined to ~50% after 6 months and about 40% at 12 months for smartphone app and smartwatch use but with lower use of the digital BP cuff throughout the 12 months. First, similar to others we observed that iPhone users were more likely to complete app-based surveys than Android users. We also observed that among iPhone users, participants using digital devices (smartwatch and BP cuff) had higher app-based survey adherence compared to participants not using the digital devices. Age was the only factor that was significantly associated with use of all three components of the eFHS CVD monitoring system. Of those who agreed to participate in the eFHS, older participants (age ≥55 years) were more likely to engage with eFHS components longer. Distinct factors were associated with engagement with each component of our system. Female sex and higher education were associated with higher completion of smartphone app surveys, whereas lower than excellent self-rated health and higher depressive symptoms were associated with lower smartwatch use. These results help to identify subgroups that may benefit from additional support to maintain long-term use of digital devices in future clinical studies and population based remote monitoring for clinical purposes. For example, with this knowledge researchers and clinicians can begin to understand the barriers to adherence and formulate strategies to assist and motivate persons with higher depressive symptoms and lower self-rated health status to maintain engagement and avoid attrition.

There are several unique aspects of our study. Participants’ attrition is a common issue in digital studies. However, our study had longer follow up time and with the sufficient data, we were able to investigate barriers of sustained device use in an integrated system. A large investigation of eight different digital health studies of a range of diseases in more than 100,000 participants demonstrated a median participant retention of about 6 days with a range of 2–26 days^20^ highlighting the importance of understanding factors associated with use of digital technologies. Among cardiovascular research studies, few deployed an integrated system that incorporated several digital devices^7,9,21^ and while the studies observed early user disengagement they did not report on factors contributing to long-term use of the digital system components^9^. Unlike our study, many digital health studies target specific health conditions^12,14^, which may limit the ability to generalize findings to the broader population. Predictors of use of wearable devices and digital health behaviors from national survey data are limited by self-report^22,23^ in contrast to our study that recorded real world transmission of data from the actual digital devices over time. Our study uses data collected with standard protocols and attempts to address these knowledge gaps by investigating a range of sociodemographic and health related factors associated with sustained use of an integrated system for digital device data collection.

Compared to previous studies, eFHS participants demonstrated considerably higher retention, that is, a substantial number of participants completed surveys and contributed smartwatch and BP data at the 12-month follow up. The participants in eFHS were not financially incentivized for participation in the study. Receiving positive notifications such as “Thank you for completing all your surveys. Your contribution is a vital part in our ongoing research efforts!” may have encouraged participants’ engagement. In addition, the study design may explain the higher retention among eFHS participants. The eFHS is nested in FHS. Participants in eFHS have been followed for more than a decade (enrolled in FHS 2002–2005). The FHS consists of loyal participants with a strong connection with the research staff that could potentially influence overall adherence rates^24^. However, the longstanding connection with the FHS may not influence any of the factors associated with digital device use.

We observed that older adults (age ≥ 55 years) were more likely to return smartphone surveys, wear the smartwatch, and send weekly BP measurements. While older adults (age 60 and older) are the least represented in digital health studies, consistent with our results, older age has been previously reported to be associated with longer retention (about 4 days in one large meta-analysis)^20^. In a cohort of individuals who self-identified as interested in digital health technologies, older age was associated with longer-term use defined as using the study device for 26 weeks^25^. Recently, the Michigan Predictive Activity & Clinical Trajectories in Health (MIPACT) study discussed results for the first 90 days of the study and included adults age 65 years and older (*n* = 1153). The study compared heart rate, step count and home BP measurements across different demographics and clinical phenotypes. However, engagement was not reported separately by age. Over the past decade, technology use has grown remarkably among older adults (aged 65 and older) including the use of smartphones and tablets^26^. Despite the progress toward closing the technology age gap, national survey data indicate that digital health engagement behaviors and use of wearables remains lower in older adults^22,23^. Among older Framingham participants (age ≥65 years) who attended exam 3, 64% declined enrollment in eFHS compared with 42% <65 years. Thus, older participants are less likely to enroll in the eFHS compared to middle age adults (Supplementary Table 2). However, once enrolled in the eFHS, older participants had higher retention and were more likely to send device data at the 12-month follow-up. Previous work also reported that technology adoption was lower for older adults but once they join the online world, digital technology often becomes an integral part of their daily lives^27^. Older adults approach to consumer health technologies to manage their health may be context dependent and change with health status, motivation, and personal health habits^28^. More research is needed to fully understand older adults’ perceptions of using technology. Younger adults in eFHS are more likely to be employed full-time compared to older adults (Supplementary Table 14) and they may not have time to engage in a long-term research study given the competing demands of a busy working life^29^. Further younger adults may have additional family obligations that result in lower engagement as a lack of time for households who are living with children was identified as a major barrier to engagement^25^.

We observed that women and participants with higher levels of education (college or professional degree) were more likely to complete eFHS smartphone app-based surveys. Our findings are consistent with observations from the Health eHeart Study that reported participants who completed survey data were more likely to be female and have a college education compared to the general US population^7^. Similarly, among individuals with CVD or diabetes, mobile health app users were more likely to be women and higher educated than non-users^30^. A possible explanation could be that women might be more concerned about their health and lifestyles^31,32^. People with higher education levels may have the skills, health literacy and confidence to use mobile devices compared to people with lower levels of education or no education^33^, but further research is necessary to assess other potential barriers to adherence to digital health studies among different demographic groups.

Depressive symptoms and self-reported health were factors associated with long-term use of the smartwatch. Studies have found a strong relationship between motivation and depressive symptoms^34,35^, and depressive symptoms are inversely associated with health-promoting lifestyle behaviors^36^. Therefore, participants with higher depressive symptoms scores may be less engaged and less likely to use a smartwatch for activities such as tracking daily steps. We observed that eFHS participants who reported excellent health were more likely to use the smartwatch throughout the 1-year period. Participants who report feeling healthier are more likely to use wearable devices^22^. Others have shown that the presence of chronic conditions is associated with lower odds of long-term use of wearable activity trackers^12^. These highlighted health disparities and sociodemographic factors could inform targeted intervention strategies to improve use of digital technologies in the future.

Apple iOS users tend to be tech-savvy and receptive to retail mobile apps^37^. Therefore the type of phone itself may be a proxy for socioeconomic status including education levels and other factors that may be associated with device use^38^. Among eFHS participants compared to iPhone users, Android users were less likely to be women, had lower education levels, and lower proportion reporting excellent health (Supplementary Table 15). Hence, Android users may need additional technologic support and other ongoing strategies to enhance long-term engagement in this study. In addition, we noted that among iPhone users, participants who use digital devices were more likely to complete app-based surveys compared to those who use only the smartphone app. A national survey reported that individuals with higher levels of technology self-efficacy were more likely to adopt and use wearables to track their health^22^. Participants who took digital devices in eFHS may be more comfortable with new technologies.

This study has several strengths. eFHS is a nested study within FHS allowing investigation of diverse factors that influence long-term use of each system component using data collected in a standardized way. Compared to other digital studies, we did not focus on a specific chronic health condition and we did not provide financial incentives to enroll participants, which helps to reduce the participation bias. We collected digital data longitudinally over 1 year, whereas many studies had shorter follow up periods. This passive monitoring allows us to report the system use over one year and evaluated factors that contribute to long-term use of an integrated system comprised of a smartphone app, smartwatch and BP cuff.

However, our study has several limitations. eFHS participants owned a smartphone, primarily resided in the New England region of the United States and they were more likely to be white, well educated, and reported excellent health (Supplementary Table 2). Thus, the findings may not be generalizable to more diverse samples. We directed participants to send BP measurements once per week and wear the smartwatch daily. Stable wireless internet connection is critical to synchronize devices with smartphone and transfer all data to secure cloud. Thus, connectivity needs to be evaluated in an ongoing manner as connectivity issues may affect device adherence overtime. The smartwatch battery required charging that may have impacted wear time. Only iPhone users were eligible to use the smartwatch and BP cuff, limiting our ability to examine engagement for Android users beyond the smartphone app. We excluded participants who enrolled in the eFHS after January 31, 2019 as a full 12 months of follow up data were not available. A few factors including alcohol consumption and current smoking status were based on self-reported answers from participants. Therefore, recall bias potentially may have affected our findings. However, we believe this to be minimal as participants were asked to report alcohol use if the consumed an alcoholic beverage at least once per month and if yes at least once per week. In this observational study, we investigated the association between each characteristic and sustained use of system components, there may be other factors associated with use that were not investigated. We did not evaluate system factors such as smartphones lost or replaced during the study or technical support provided. We cannot eliminate residual confounding and we cannot establish any causal relationship between characteristics and use of digital devices.

Among middle-aged to older adult participants of the eFHS, sociodemographic and health related factors were associated with use over 1 year of an integrated digital CVD monitoring system with a smartphone app, smartwatch, and digital BP cuff. The findings of this study can be used to plan future research to increase engagement when deploying digital devices for CVD monitoring. Participants with lower educational levels, lower than excellent self-reported health, and higher depressive symptoms may benefit from additional support to enhance sustained device use. Self-disease management will be more prevalent in the future and to avoid a digital divide, various stakeholders need to focus on these groups who are at risk of attrition when developing strategies to improve engagement. In addition, there should be resources for these groups to improve health literacy and computer/phone literacy. Given differences in engagement among demographic groups, it may also be useful to use community engagement strategies to reduce attrition.

## Methods

### Study sample and eFHS system components

Participants were recruited from the FHS Third Generation (Gen 3) cohort (*n* = 4095), multiethnic Omni Group 2 Cohort (*n* = 410) and New Offspring Spouse (*n* = 103) who were initially enrolled into the FHS from 2002 to 2005. FHS participants undergo periodic examinations every 6–8 years^39^. At the beginning of June 2016, eFHS invited participants who attended exam 3 (2016–2019) and who spoke English to enroll^18^. To be eligible, eFHS participants owned a smartphone (Android or compatible iPhone iOS version 9 or higher). All participants provided written informed consent (two steps: first step at exam 3 and electronic consent as the second step within the eFHS app) and written eFHS protocol^18^. The eFHS study was reviewed and approved by the Institutional Review Board at Boston University Medical Center.

Among 2151 participants enrolled in the eFHS, 203 individuals (who enrolled from Feb 2019 to Aug 2019) were excluded due to being followed for <12-months from enrollment (Fig. 1). In the eFHS, a smartphone app was used to collect health information using app-based surveys that were deployed at baseline and every 3 months^24^. Participants received welcome notifications at enrollment, notifications when new surveys became available, reminder notifications to complete surveys, and thank you messages after completing all surveys. eFHS participants who owned iPhones were eligible to choose to use a wireless BP cuff (Nokia/Withings) and/or a smartwatch (Apple Watch series 0). The eFHS research technician assisted with app download and device pairing at the research center or remotely. Participants were asked to measure their BP at the same time each week for up to 1 year. In November 2016, the smartwatch was introduced for monitoring participants’ step count and heart rate. Participants were instructed to wear the smartwatch daily over 1 year^18^. Participants also received reminder notifications to send BP data or watch data if they didn’t send any records within 2 weeks, thank you messages when they took their BP again after more than 2 weeks of non-compliance, and motivational message (“You’re on a Roll!”) if they provide continuous BP records in the last 2 weeks. System components were investigated separately due to the differences in the number of individuals who used the app or devices and the differences in the frequency of the use: at baseline and every 3 months for surveys on the app, daily use for the watch, and weekly use for the BP cuff. One-year follow up data were considered for the analysis of all system components.

### Measures of use of each eFHS system component

For the smartphone app, several surveys such as physical activity, alcohol consumption, depression symptoms were deployed at baseline (enrollment date) and every 3 months. In particular, 9 surveys were deployed at baseline, only one survey at 3 months, 4 surveys at 6 months, 1 survey at 9 months, and 7 surveys at 12 months (Supplementary Table 1). Copy of all survey questions were also provided (Supplementary Table 1). The smartphone app surveys are derived from the questionnaires used in the FHS Research Center and surveys to obtain medical history update information between FHS Research Center examinations. Most of these questionnaires are available on the FHS website. More information of how the survey was developed and pre-tested was previously reported^24^. We considered a survey completed when participants completed 75% of the survey questions. We use two definitions for survey app use (Supplementary Table 3). The first definition identified participants who completed at least one survey at baseline and each 3-month window, and the second definition identified participants who completed all surveys at a given survey wave^24^. The use of a BP cuff was defined as sending at least one BP measurement in a given week (Supplementary Table 3). Smartwatch use needs to be evaluated in terms of smartwatch wear time that was determined based upon the presence of step or heart rate data. As previously reported, a valid study day in eFHS was defined as days in which participants’ wear time was ≥5 h per day^40^. This helps minimize the bias caused by no or low smartwatch wear time. Smartwatch use was defined if participants wore the watch for more than 5 h at least one day of the week^41^.

### Candidate predictors of use of eFHS system components

A set of predictors from several domains were chosen because of potential to affect adherence to mobile health devices and recent report of association with self-reported device use^22^ (Supplementary Fig. 2). We examined the same set of 18 factors for association with use of each system component over the 12-month study period. The key predictor variables of interest include several socio-demographic factors^42^ and health-related factors^43^ (Supplementary Fig. 2). Factors were obtained at the FHS in-person exam 3 using standard protocols.

Sociodemographic factors included self-reported age, sex, marital status, race/ethnicity, education, and employment status. Participants who reported marital status as “Married/living as married/living with partner” were considered as married individuals. We categorized education into four groups: graduate or professional degrees, completed bachelor’s degree, completed some college (some college but no degree, completed technical school certificate or associate degree) and completed high school or less than high school. Full time employment was classified as a binary variable: participants who reported their current employment as full time employment or full time self-employment vs. not.

Lifestyle factors included smoking, alcohol, and physical activity. Current smoking was defined as smoking cigarettes regularly (≥1 cigarette a day for 1 year) in the last year. For those who responded that they consumed any alcohol, the average amount of grams in beer, wine, and liquor/spirits was multiplied by the number of drinks per week. This measure was considered as grams consumed per day and current alcohol consumption was dichotomized based on grams consumed per day >0. Physical activity index was computed as a composite score of hours spent for sleep, sedentary, slight, moderate, and heavy physical activities with corresponding weights equal to 1, 1.1, 1.5, 2.5, and 5^44^. Subjective health was assessed with the question “In general, would you say your health is: Poor, Fair, Good, Very Good, Excellent.” “Poor” and “Fair” were collapsed into one category for analyses^45^. The 20-item Center for Epidemiologic Studies-Depression scale was used to assess depressive symptoms based on scores calculated by summing up all answers for 20 questions^46^.

CVD risk factors and health conditions included body mass index (BMI), cholesterol (mg/dL), systolic blood pressure (SBP, mmHg), diastolic blood pressure (DBP, mmHg), hypertension, diabetes mellitus, and prevalent CVD (defined below). Cholesterol levels were used together with reports of use of lipid lowering medication. Average value of first and second readings of SBP or DBP were considered for the analysis. SBP or DBP were considered together with reports of hypertensive medication use. Hypertension was defined as self-report of medication use to treat high BP or hypertension and/or BP measurement (SBP ≥ 140 or DBP ≥ 90). Diabetes was characterized by fasting blood glucose level ≥126 mg/dL or current use of any blood glucose-lowering medications. Prevalence of CVD was defined as participants who had coronary heart disease (including myocardial infarction, coronary insufficiency, and angina), heart failure, stroke, and intermittent claudication^47^. Events were adjudicated by a panel of investigators using standard criteria. We found missing values for several variables including employment status, marital status, diabetes, cholesterol level, BMI, and subjective health (See footnote of Table 1). If data were missing for a variable at exam 3, we used data from a prior attended exam.

### Statistical analysis

Characteristics were considered across each digital device. Mean (standard deviation) were reported for continuous variables and counts (percentages) were reported for categorical variables. Proportion of participants who complete at least one surveys and all surveys stratified by sex were plotted across each 3-month intervals based on empirical values. Proportions of participants who wore the watch for more than 5 h at least 1 day of the week and proportions of participants who sent BP data once per week were calculated across each week and plotted on the same graph. As an exploratory data analysis, we were interested in examining smartwatch use and BP use stratified by different age groups based on empirical values. Thus, age was categorized into four different age groups (age <45, 45 ≤ age <55, 55 ≤ age <65, and age ≥65 years) after careful inspection of the distribution of age. Proportions of participants who used the watch (wear for ≥1 day for ≥5 h per day) and BP cuff (BP measurement returns ≥1 per week) stratified by these age groups were plotted across each week.

Longitudinal data was considered when we investigated the association between each predictor and digital device use. The following statistical analysis workflow (Supplementary Fig. 3) was repeated for each system component using 12-month follow up data. To be consistent with the exploratory analysis, the same age groups were considered in statistical models. A generalized linear mixed model (GLMM) was fitted per each factor listed in Supplementary Fig. 2 adjusting for the above age groups, sex, and time of use (weeks for digital device use and 3-month period for app use). The GLMMs for each factor were examined and selected as individual predictors associated with device use or app use if *P* < 0.1 (P value derived using *Z* test statistic from GLMMs) significance level was met. Individual predictor testing does not consider the confounding effect of other factors. Weak predictors in the individual association testing may have strong associations when considered together with other factors^48^. To address this issue, we considered a relaxed threshold (*P* < 0.1, *P* value derived using *Z* test statistic from GLMMs) to prioritize individual predictors in the multivariable model^49–52^. Participants’ IDs were included as a random effect in GLMM to capture the correlation among repeated measures from the same participant. All continuous variables were scaled to mean of 0 and standard deviation of 1 to avoid convergence issues in the GLMM. Time of use was treated as an ordinal variable (baseline, 3-month, 6-month, 9-month, and 12-month) for app survey analysis and continuous variable (weeks) for analysis of BP cuff and Watch use. Next, a multivariable model for each system component was considered with all predictors selected from initial testing adjusting for age, sex, and time of use. To define statistical significance in the final multivariable models, *p* < 0.05 (*P* value derived using *Z* test statistic from GLMMs) was used.

As secondary analysis, we investigated whether age and time of use are effect modifiers in the multivariable models using following statistical analysis workflow (Supplementary Fig. 4). First, we investigated whether effect of age and sex in the final multivariable model differ according time of use. Separate GLMM was fitted including each interaction terms; Age × time of use, and sex × time of use. Stratified analyses were performed for all three-system components according to usage time. In the existing literature, individuals were categorized as long term users if their usage was 6 months or more^12^. Thus, stratified analyses were conducted for weeks ≤26 and weeks >26 to investigate effect modification with respect to time of digital device use. Separate analyses were conducted for app use in each 3-month period. Next, we investigated whether effect of each predictor in the final multivariable model differ according to age groups. The interaction between participant characteristics and age groups were included in the final multivariable models. We considered stratified analysis with respect to age, we considered two groups; age ≤53 and age >53 years (median age 53).

To further investigate whether there was a linear association of BP cuff and watch use, the correlation coefficient of BP cuff and watch use was computed by considering subgroups of participants who took both devices. All statistical analyses were performed with R software (R version 4.0.5) and the lme4 package in R was used for analysis with GLMMs.

### Reporting summary

Further information on research design is available in the Nature Research Reporting Summary linked to this article.

## Supplementary information

Supplementary Information

Reporting Summary

## Data Availability

Data of the eFHS will be available at the Biologic Specimen and Data Repository Information Coordinating Center (BioLINCC). BioLINCC has a 2-year release policy but data will be accessible to investigators via the authors until released by BioLINCC. Requests for the code used in this study should be made to the corresponding author.

## Code availability

Code for the analysis is available upon reasonable request from the corresponding author.
