# Supplementary material for: Factors associated with long-term use of digital devices in the electronic Framingham Heart Study
Source: NPJ Digit Med. 2022 Dec 27;5:195. doi: 10.1038/s41746-022-00735-1 (PMC9792462; doi:10.1038/s41746-022-00735-1)
Supplement: Supplementary file 1 — Supplementary Information [file 41746_2022_735_MOESM1_ESM.pdf]

Supplementary Information

Factors Associated with Long-term Use of Digital Devices in the Electronic Framingham Heart Study

Chathurangi H Pathiravasan* [1], Yuankai Zhang[1], Xuzhi Wang[1], Ludovic Trinquart[1,2,3], Emelia J Benjamin[4,5], Belinda Borrelli[6], David D McManus[7,8], Vik Kheterpal[9], Honghuang Lin[7], Nicole L Spartano[10], Eric Schramm[9], Chunyu Liu[1], Joanne M Murabito[4,11]

1. Department of Biostatistics, Boston University School of Public Health, Boston, MA, USA
2. Institute for Clinical Research and Health Policy Studies, Tufts Medical Center, Boston, MA, USA
3. Tufts Clinical and Translational Science Institute, Tufts University, Boston, MA, USA
4. Boston University's and National Heart, Lung, and Blood Institute's Framingham Heart Study, Framingham, MA, USA
5. Section of Cardiovascular Medicine, Department of Medicine, Boston Medical Center, Boston University Schools of Medicine and Department of Epidemiology, Boston University School of Public Health, Boston, MA, USA
6. Henry M. Goldman School of Dental Medicine, Center for Behavioral Science Research, Department of Health Policy & Health Services Research, Boston University, Boston, MA, USA
7. Department of Medicine, University of Massachusetts Chan Medical School, Worcester, MA, USA
8. Department of Quantitative Health Sciences, University of Chan Massachusetts Medical School, Worcester, MA, USA
9. Care Evolution, Ann Arbor, MI, USA
10. Section of Endocrinology, Diabetes, Nutrition, and Weight Management, Boston University School of Medicine, Boston, MA, USA
11. Section of General Internal Medicine, Department of Medicine, Boston University School of Medicine and Boston Medical Center, Boston, MA, USA

* Corresponding author: chathurangi.heshani@gmail.com

Table of Contents

Supplementary Figure 1. Venn diagram of participants who returned surveys, blood pressure cuff measurements and smartwatch data 1
Supplementary Figure 2. Domains and individual factors hypothesized to be associated with the use of eFHS system components 2
Supplementary Figure 3. Primary Analysis: Analysis Workflow. Note: Here * refer to each predictors listed in the Figure 2 in the manuscript, ** refer to time of use (weeks for device use and 3-month period for app use). P values are derived using Z test statistic from generalized linear mixed models (GLMM) 3
Supplementary Figure 4: Secondary Analysis: Analysis Workflow. Note: Here * refer to time of use groups (mobile app use: 3-months intervals, Smartwatch & BP Cuff use: weeks ≤ 26 and weeks > 26) and ** refer to age groups: age ≤ 53 and age > 53 years (median age 53). P values are derived using Z test statistic from generalized linear mixed models (GLMM) 4
Supplementary Figure 5. Correlation of Smartwatch Use and Digital BP Cuff Use 5

Supplementary Table 1. eFHS Baseline and Follow-up Survey Components and All survey questions 6
Supplementary Table 2: Characteristics of participants in eFHS Participants vs those who did not enroll in the eFHS 21
Supplementary Table 3. Definitions of Smartphone App Use, Smartwatch Use, and Digital BP Cuff Use 22
Supplementary Table 4. Predictor variables selection for final multivariable model of smartphone app use (completing at least one survey) 23
Supplementary Table 5. Predictor variables selection for final multivariable model of smartphone app use (completing all surveys) 25
Supplementary Table 6. Stratified analysis with smartphone app use of each 3- month period 27
Supplementary Table 7. Stratified analysis for app use according to different age groups 28
Supplementary Table 8. Predictor variables selection for final multivariable model of smartwatch use 29
Supplementary Table 9. Predictor variables selection for final multivariable model of digital blood pressure cuff use 31
Supplementary Table 10. Stratified analysis with smartwatch use time (Weeks ≤ 26 and Weeks > 26) 33
Supplementary Table 11. Stratified analysis with digital blood pressure cuff use time (Weeks ≤ 26 and Weeks > 26) 34
Supplementary Table 12. Stratified analysis for smartwatch use according to different age groups (Age ≤ 53 and Age > 53) 35
Supplementary Table 13. Stratified analysis for digital blood pressure cuff use according to different age groups (Age ≤ 53 and Age > 53) 36
Supplementary Table 14. Characteristics of eFHS Participants stratified according different age groups 37
Supplementary Table 15. Characteristics of eFHS Participants stratified according type of phone 38

Supplementary Figure 1. Venn diagram of participants who returned surveys, blood pressure cuff measurements and smartwatch data

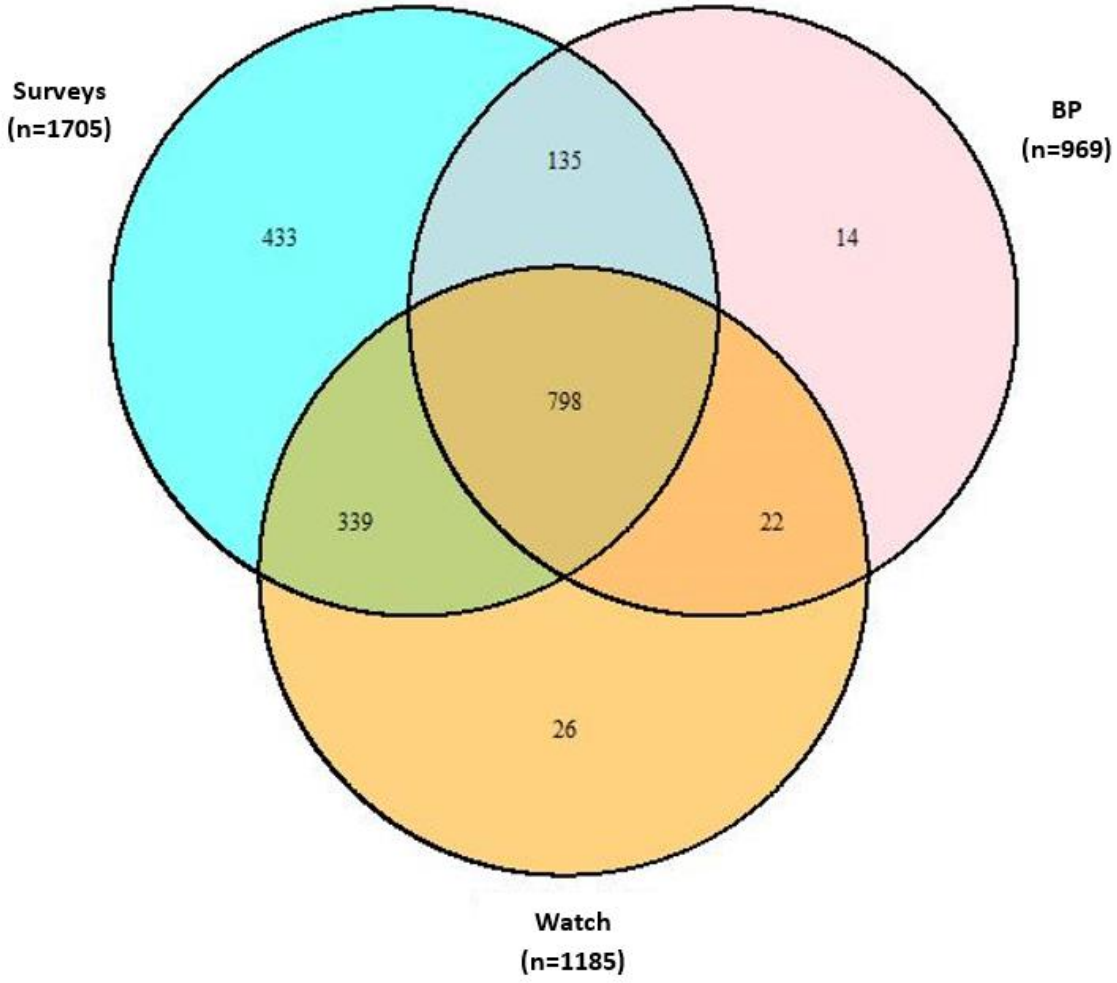

Supplementary Figure 2. Domains and individual factors hypothesized to be associated with the use of eFHS system components

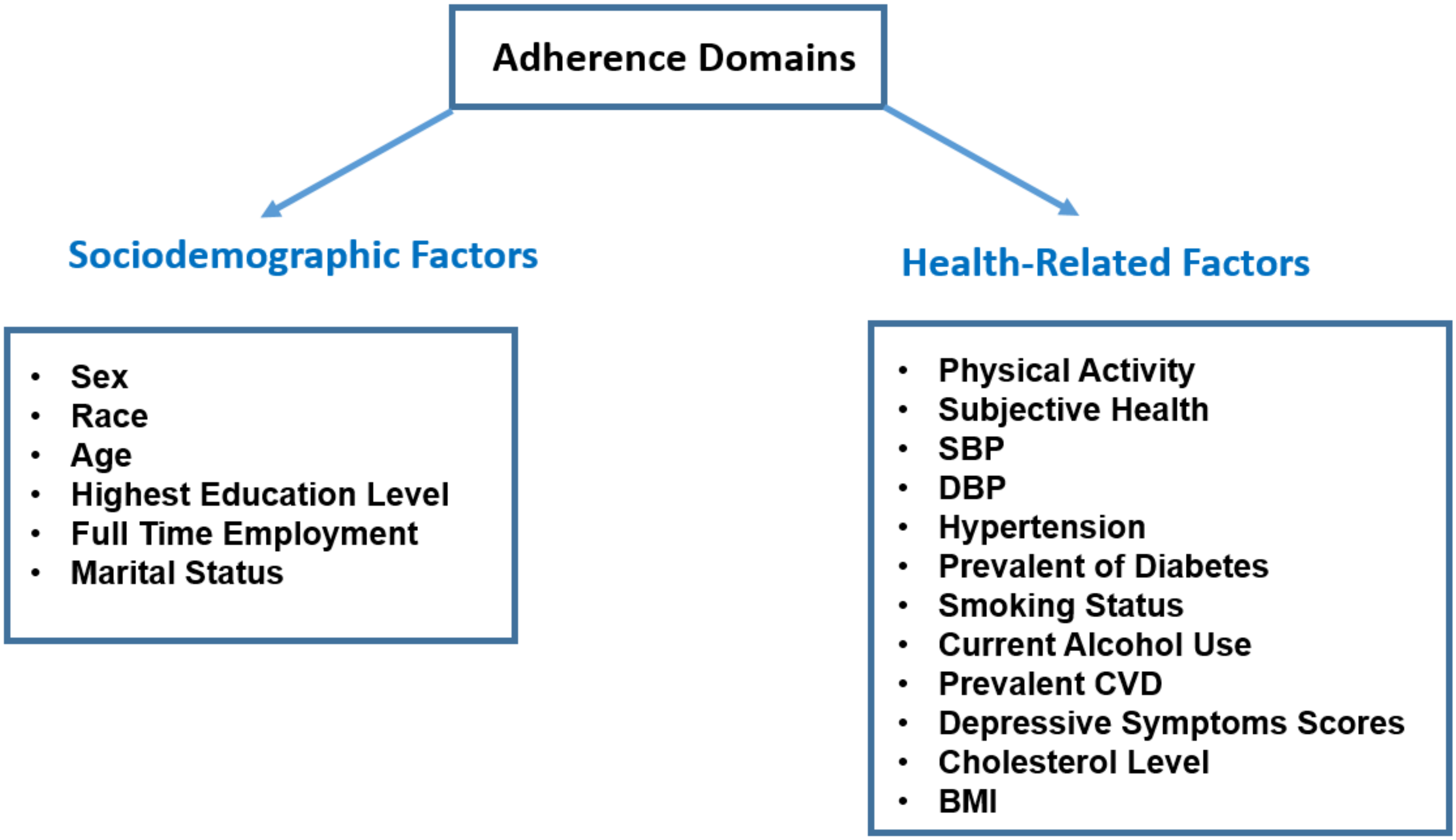

Supplementary Figure 3. Primary Analysis: Analysis Workflow. Note: Here * refers to each predictor listed in Figure 2 in the manuscript, ** refers to time of use (weeks for device use and 3-month period for app use). P values are derived using Z test statistic from generalized linear mixed models (GLMM)

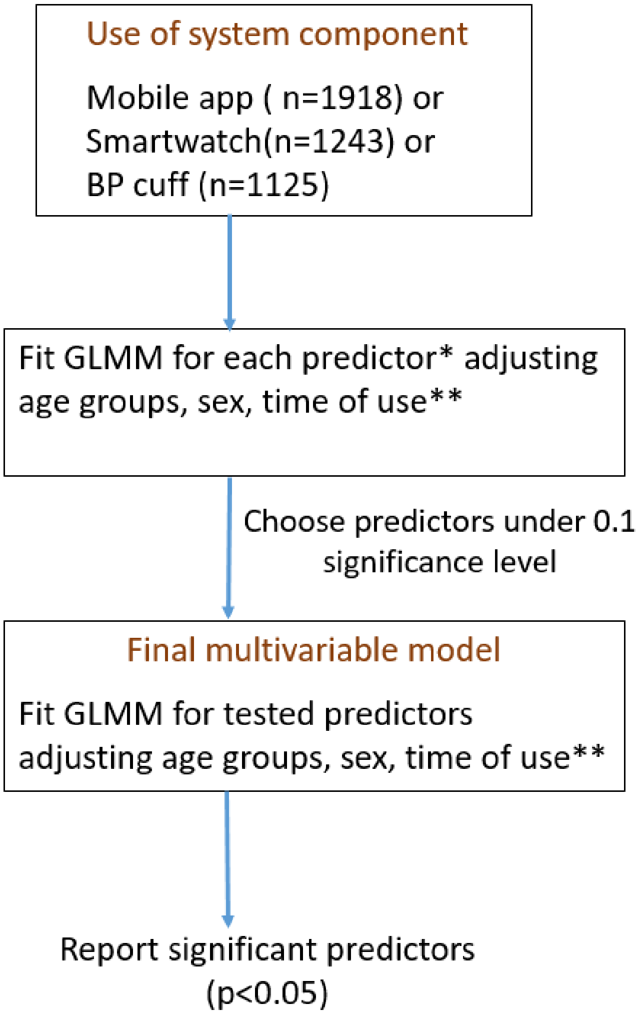

Supplementary Figure 4: Secondary Analysis: Analysis Workflow. Note: Here * refers to time of use groups (mobile app use: 3-months intervals, Smartwatch & BP Cuff use: weeks ≤ 26 and weeks > 26) and ** refers to age groups: age ≤ 53 and age > 53 years (median age 53). P values are derived using Z test statistic from generalized linear mixed models (GLMM)

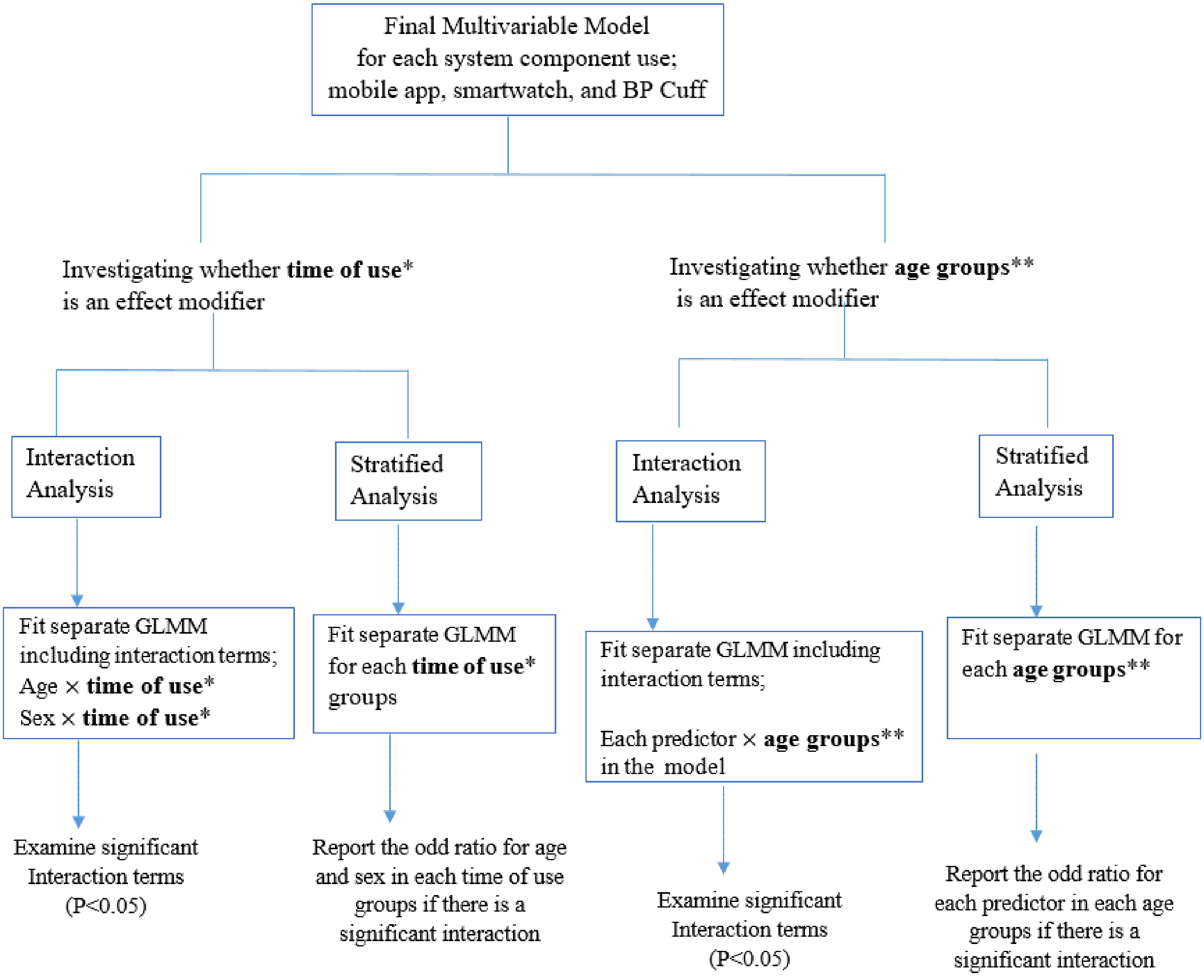

Supplementary Figure 5. Correlation of Smartwatch Use and Digital BP Cuff Use

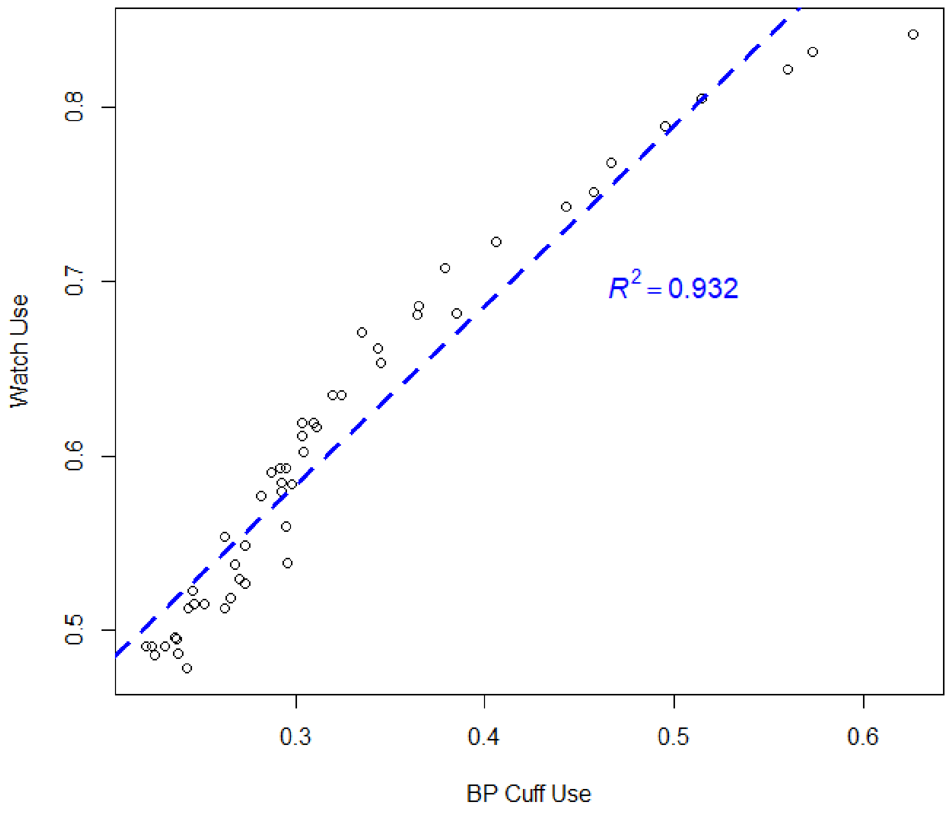

Supplementary Table 1. eFHS Baseline and Follow-up Survey Components and All survey questions

eFHS Baseline and Follow-up Survey Components

Time of Survey Deployment	Surveys Deployed
**Baseline***	1. Socio-demographics<br>2. Medications and self-reported risk factors<br>3. Smoking<br>4. Alcohol use<br>5. Physical activity<br>6. Depressive symptoms (CES-D)<br>7. Health Survey<br>8. Baseline CVD history<br>9. Baseline non-CVD Medical history
**3 months***	1. Physical activity
**6 months***	1. Medical history update<br>2. Physical activity<br>3. Depressive symptoms (CES-D)<br>4. Health Survey
**9 months***	1. Physical activity
**12 months***	1. Medical history update<br>2. Medications and self-reported risk factors<br>3. Physical activity<br>4. Depressive symptoms (CES-D)<br>5. Health Survey<br>6. Smoking<br>7. Alcohol intake

All survey questions

- ### Socio-demographics

1. "What is your current marital status?

   Single, never married
   Married
   Separated
   Divorced
   Widowed
   Living with partner
   Prefer not to answer

2. "What is the HIGHEST degree or level of school you have completed? (Select one) If you are currently enrolled, select the highest grade you have completed or the highest degree you have received."

   No schooling
   Grades 1-8
   Grades 9-11
   Completed high school (12th grade) or GED
   Some college but no degree
   Technical school certificate
   Associate degree (Junior college AA, AS)

Bachelor’s degree (BA, AB, BS)
Graduate or professional degree (master’s, doctorate, MD, etc.)
Prefer not to answer

3. Please choose which of the following best describes your current employment status. (Select one)

   Homemaker, not working outside the home
   Employed (or self-employed) full time
   Employed (or self-employed) part time
   Employed, but on leave for health reasons
   Employed, but temporarily away from my job
   Unemployed or laid off
   Retired from my usual occupation and not working
   Retired from my usual occupation but working for pay
   Retired from my usual occupation but volunteering
   Prefer not to answer
   Unemployed due to disability
   Full-time student

4. What is your current occupation? Please select the response that best describes your occupation.

   homemaker
   retired
   self-employed business owner
   M.D./dentist
   lawyer/judge
   psychologist/social worker/mental health counselor
   scientist/research
   engineer/computer science
   banker/accountant
   manager/consultant (e.g. production manager)
   administrative (e.g. personnel)
   educator
   nurse/medical personnel
   laboratory technician
   physical/occupational/speech therapist
   secretary/clerk/data entry
   retail/cashier
   sales/marketing/insurance
   realtor
   writer/editor
   artist/graphic designer/craftperson

5. Please select the income group that best represents your combined family income for the past 12 months. (Select one)
   Under $20,000
   $20,000 – $34,999
   $35,000 – $54,999
   $55,000 – $74,999
   $75,000 – $100.000
   Over $100,000
   Prefer not to answer

6. How many people are supported by this income?

7. What form of health insurance do you have currently? (Select ALL that apply)

   Blue Cross Blue Shield
   Harvard-Pilgrim
   Tufts
   Aetna
   United Health Care
   Medicare
   Medicaid
   Military or Veteran's administration sponsored
   Other
8. Do you have prescription drug coverage?

- **Alcohol use**

1. Do you drink beer at least once a month?
2. Average number of servings of beer (12oz bottle, glass, can)
3. Do you drink wine at least once a month?
4. Average number of servings of wine (red or white, 4oz glass)
5. Do you drink liquor/spirits at least once a month?
6. Average number of servings of liquor/spirits (1oz cocktail/highball)
7. At what age did you stop drinking alcohol?
8. Over the past year, on average on how many days per week did you drink an alcoholic beverage of any type?
9. Over the past year, on a typical day when you drink, how many drinks do you have?
10. What was the maximum number of drinks you had in a 24 hour period during the past month?
11. Since your last FHS examination, has there been a time in your life when you drank 5 or more alcoholic drinks of any kind almost daily?

- **Medications and self-reported risk factors**

1. In the PAST YEAR, have you been told by the doctor that you have any of the following conditions? (Select ALL that apply)

   High blood pressure or hypertension
   High blood cholesterol or high triglycerides
   High blood sugar or diabetes
   None of the above

2. In the PAST YEAR, have you taken medication for any of the following conditions? (Select ALL that apply)

   High blood pressure or hypertension
   High blood cholesterol or high triglycerides
   High blood sugar or diabetes
   Cardiovascular disease (for example angina/chest pain, heart failure, atrial fibrillation/heart rhythm abnormality, stroke, leg pain when walking, peripheral artery disease)

None of the above

3. Do you take aspirin regularly?
4. How many aspirins do you take regularly?
5. How often do you take aspirin?
6. What is your usual dose of aspirin?
7. The following questions ask about physical and laboratory measurements for evaluating CVD risk.
8. What is your most recent weight?
9. What is your most recent height?
10. What is your most recent blood pressure?
11. What is your most recent total cholesterol level?
12. What is your most recent HDL level?
13. What is your most recent LDL level?

- **Smoking**

1. Have you ever smoked tobacco cigarettes?
2. Have you smoked cigarettes regularly in the last year? (No means less than 1 cigarette a day for 1 year.)
3. Do you now smoke cigarettes (as of 1 month ago)?
4. How many cigarettes do you smoke per day now?
5. On the average of the entire time you smoked, how many cigarettes did you smoke per day?
6. How old were you when you first started regular cigarette smoking?
7. If you have stopped smoking cigarettes completely, how old were you when you stopped?
8. When you were smoking, did you ever stop smoking for >6 months?
9. For how many years in total did you stop smoking cigarettes? (If 6 months to 1 year, enter "1")
10. Since your last exam, have you regularly smoked a pipe or cigar?
11. Do you smoke a pipe or cigar now?
12. E-cigarettes are battery-powered and produce vapor instead of smoke. Have you ever tried an e-cigarette?
13. Have you ever been a regular user of e-cigarettes (at least once per week)?
14. How long did you use e-cigarettes?
15. How many days per week, on average, did you use e-cigarettes while you were a regular user?
16. In the past 5 days, including today, on how many days did you smoke an e-cigarette?

- **Baseline CVD history**

1. Since the date of your last FHS examination (exam 2, several years ago), have you seen a doctor or been hospitalized?
2. Did you have any of the following heart problems? (Select ALL that apply)

   Chest pain, angina or angina pectoris
   Heart attack or myocardial infarction or MI
   Heart failure or congestive heart failure or CHF
   Atrial fibrillation or atrial flutter
   Other heart problem (valve problem, ventricular tachycardia, other rhythm problem)
   None of the above

3. Please specify any other heart problems that weren't captured in the list.
4. Did you have any of the following heart procedures? (Select ALL that apply)

   Heart catheterization or cardiac catheterization
   Heart bypass operation or coronary bypass surgery or CABG
   Procedure to unblock narrowed blood vessels to your heart muscles (PTCA, coronary angioplasty, or coronary stent)
   Other heart procedure (pacemaker, aorta surgery)
   None of the above

5. Please specify any other heart procedures that weren't captured in the list.
6. Did you have any of the following circulatory problems? (Select ALL that apply)

   Stroke, TIA (transient ischemic attack), sudden paralysis, vision loss, inability to speak
   Procedure to unblock narrowed blood vessels in your neck (carotid endarterectomy, carotid angioplasty)
   Poor blood circulation or blocked or narrowed blood vessels to the legs or feet, (claudication, peripheral arterial disease, gangrene)
   Amputation of part of a leg or toes, because of poor circulation or gangrene
   Blood clot or embolism in leg or lung
   Other circulatory problem
   None of the above"

7. Please specify any other circulatory problems that weren't captured in the list.

- **Baseline-Non-CVD Medical History**

1. Have you ever been told that you have any of the following endocrine conditions? (Select ALL that apply)

   Thyroid disease
   Diabetes mellitus
   Other kinds of endocrine diseases
   None of the above

2. Please specify any other endocrine diseases
3. Have you ever been told that you have any of the following genitourinary or gynecologic conditions? (Select ALL that apply)

Urinary infection
Chronic kidney disease
Other kidney disease
Prostate disease
Gynecologic problems
None of the above

4. Have you ever been told that you have any of the following genitourinary or gynecologic conditions? (Select ALL that apply)

   Urinary infection
   Chronic kidney disease
   Other kidney disease
   None of the above

5. Have you ever been told that you have any of the following genitourinary conditions? (Select ALL that apply)

   Urinary infection
   Chronic kidney disease
   Other kidney disease
   Prostate disease
   None of the above

6. Please specify the type of kidney disease

7. Please specify the type of gynecologic problems

8. Have you ever been told that you have any of the following pulmonary conditions? (Select ALL that apply)

   Emphysema
   Pneumonia
   Asthma
   Sleep Apnea
   Other kind of lung disease
   None of the above

9. Please specify the type of lung disease

10. Have you ever been told that you have any of the following rheumatologic conditions? (Select ALL that apply)

    Gout
    Degenerative joint disease, or osteoarthritis
    Rheumatoid arthritis
    Other muscle, joint, or connective tissue diseases
    None of the above

11. Please specify the type of muscle, joint, or connective tissue disease

12. Have you ever been told that you have any of the following gastrointestinal conditions? (Select ALL that apply)

    Gallbladder disease
    Gastric bypass surgery or a lap band

GERD or ulcer disease
E_LIVER Liver disease
Other GI diseases
None of the above"

13. Please specify the type of gastrointestinal disease

14. Have you ever been told that you have any of the following mental health conditions? (Select ALL that apply)

    Depression
    Anxiety
    Other mental health diseases
    None of the above

15. Please specify any other mental health diseases

16. Have you ever been diagnosed with cancer?

17. Please specify the type of cancer

18. Have you ever been told that you have any of the following other conditions? (Select ALL that apply)

    Eye disease
    Disease of your ear, nose, or throat
    Skin diseases
    Heart murmur
    Blood disorder
    Bleeding disorder
    Any infectious diseases
    Any other diseases
    None of the above

19. Please specify the type of eye disease

20. Please specify the type of disease of your ear, nose, or throat

21. Please specify the type of skin disease

22. Please specify the type of blood disorder

23. Please specify the type of bleeding disorder

24. Please specify the type of infectious diseases

25. Please specify any other diseases.

- **Physical Activity**

1. Are you able to walk half a mile without help? (About 4-6 blocks)

2. Rest and activity for a typical day over the past year. (A typical day = most days of the week) Activities must equal 24 hours.

3. How many hours per night do you typically sleep?

4. How many hours per day are you typically sitting?

5. How many hours per day do you spend in light activities such as standing or walking?

6. How many hours per day do you spend in moderate activity including housework (vacuum, dusting), yard chores, climbing stairs; light sports such as bowling, golf?

7. How many hours per day do you spend in heavy activity including heavy household work, heavy yard work such as stacking or chopping wood, or exercise such as intensive sports--jogging, swimming etc.?

8. The next questions are about your leisure time. In the past week, about how many hours per day did you sit and watch TV or videos?

   None or <1 hour
   1 hour
   2 hours
   3 hours
   4 hours
   5 hours or more

9. In the past week, about how many hours per day did you use a computer or play computer games or play video games?

   None or <1 hour
   1 hour
   2 hours
   3 hours
   4 hours
   5 hours or more

10. The next questions are about physical activities in the past 24 hours that may not have been captured by your phone or wearable device.

11. In the last 24 hours, how often did you have your phone or wearable device with you?

    All day and all night
    All day, but not at night
    About half of the time
    Rarely if at all

12. Did you perform any physical activities yesterday that you think were not recorded by your phone or wearable device?

13. Which activity did you do that may have been improperly recorded? (Select ONE. You will be able to add additional activities later.)

    Walking
    Jogging
    Cycling
    Tennis or other racquet sport
    Soccer, basketball, or other team sport
    Weight lifting
    Swimming
    Other

14. Please specify the type of other activity.

15. How long did you do the activity?

16. How intense was the activity?

17. Did you perform any additional physical activities that you think were not recorded by your phone or wearable device?

18. Which activity did you do that may have been improperly recorded? (Select ONE. You will be able to add additional activities later.)

Walking
Jogging
Cycling
Tennis or other racquet sport
Soccer, basketball, or other team sport
Weight lifting
Swimming
Other

19. Please specify the type of other activity.

20. How long did you do the activity?

21. How intense was the activity?

Light
Moderate
Vigorous

22. Did you perform any additional physical activities that you think were not recorded by your phone or wearable device?

23. Which activity did you do that may have been improperly recorded? (Select ONE. You will be able to add additional activities later.)

Walking
Jogging
Cycling
Tennis or other racquet sport
Soccer, basketball, or other team sport
Weight lifting
Swimming
Other

24. Please specify the type of other activity.

25. How long did you do the activity?

26. How intense was the activity?

Light
Moderate
Vigorous

27. Did you perform any additional physical activities that you think were not recorded by your phone or wearable device?

28. Which activity did you do that may have been improperly recorded? (Select ONE. You will be able to add additional activities later.)

Walking
Jogging
Cycling
Tennis or other racquet sport
Soccer, basketball, or other team sport
Weight lifting
Swimming
Other

29. Please specify the type of other activity.

30. How long did you do the activity?

31. How intense was the activity?

Light
Moderate
Vigorous

- **Depressive symptoms (CES-D)**

Reponses for all questions

0 - Rarely or none of the time (less than 1 day)
1 - Some or a little of the time (1-2 days)
2 - Occasionally or moderate amount of time (3-4 days)
3 - Most of or all of the time (5-7 days)

1. During the past week, I was bothered by things that usually don’t bother me.

2. During the past week, I did not feel like eating; my appetite was poor.

3. During the past week, I felt that I could not shake off the blues, even with help from my family and friends.

4. During the past week, I felt that I was just as good as other people.

5. During the past week, I had trouble keeping my mind on what I was doing.

6. During the past week, I felt depressed.

7. During the past week, I felt that everything I did was an effort.

8. During the past week, I felt hopeful about the future.

9. During the past week, I thought my life had been a failure.

10. During the past week, I felt fearful.

11. During the past week, my sleep was restless.

12. During the past week, I was happy.

13. During the past week, I talked less than usual.

14. During the past week, I felt lonely.

15. During the past week, people were unfriendly.

16. During the past week, I enjoyed life.

17. During the past week, I had crying spells.

18. During the past week, I felt sad.

19. During the past week, I felt that people disliked me.

20. During the past week, I could not “get going.”

- **Medical history update survey**

1. Since the date of when you downloaded the eFHS app/or completed your medical history update, have you seen a doctor or been hospitalized?

2. Did you have any of the following heart problems? (Select ALL that apply)

   Chest pain, angina or angina pectoris
   Heart attack or myocardial infarction or MI
   Heart failure or congestive heart failure or CHF
   Atrial fibrillation or atrial flutter
   Other heart problem (valve problem, ventricular tachycardia, other rhythm problem)
   None of the above

3. Please specify any other heart problems that weren't captured in the list.

4. Did you have any of the following heart procedures? (Select ALL that apply)

   Heart catheterization or cardiac catheterization
   Heart bypass operation or coronary bypass surgery or CABG
   Procedure to unblock narrowed blood vessels to your heart muscles (PTCA, coronary angioplasty, or coronary stent)
   Other heart problem (pacemaker, aorta surgery)
   None of the above

5. Please specify any other heart procedures that weren't captured in the list.

6. Did you have any of the following circulatory problems? (Select ALL that apply)

   Stroke, TIA (transient ischemic attack), sudden paralysis, vision loss, inability to speak
   Procedure to unblock narrowed blood vessels in your neck (carotid endarterectomy, carotid angioplasty)
   Poor blood circulation or blocked or narrowed blood vessels to the legs or feet, claudication, peripheral arterial disease, gangrene)
   Amputation of part of a leg or toes, because of poor circulation or gangrene
   Blood clot or embolism in leg or lung
   Other circulatory problem
   None of the above

7. Please specify any other circulatory problems that weren't captured in the list.

8. Did you have any of the following other problems? (Select ALL that apply)

   Diabetes
   Cancer

9. Specify type, Physician, Place where biopsy performed, select if no biopsy

10. Since the date of when you downloaded the eFHS app/or completed your medical history update, have you been admitted to a HOSPITAL or gone to an EMERGENCY ROOM or seen a PHYSICIAN for other than a routine examination? Date?

11. What type of visit did you have?

12. What was the reason for your visit? (Select ONE)

13. Please specify the reason for your visit. Hospital Name, Doctor's Name, Location (City, State)

14. Have you any other admissions to a HOSPITAL, visits to an EMERGENCY ROOM, or visits to a PHYSICIAN for other than a routine examination since the date of when you downloaded the eFHS app/or completed your medical history update? Date?

15. What type of visit did you have?

    Overnight admission
    Emergency room visit
    Day Surgery/Procedure
    M.D. visit

16. What was the reason for your visit?

    Heart problems
    Stroke or transient ischemic attack (TIA), sudden paralysis, vision loss, inability to speak
    Broken, crushed or fractured bones
    Cancer or malignant tumor
    Circulation problem, or blood clots
    Other reasons

17. Please specify the reason for your visit. Hospital Name, Doctor's Name, Location (City, State)

18. Have you any other admissions to a HOSPITAL, visits to an EMERGENCY ROOM, or visits to a PHYSICIAN for other than a routine examination since the date of when you downloaded the eFHS app/or completed your medical history update? Date?

19. What type of visit did you have?

    Overnight admission
    Emergency room visit
    Day Surgery/Procedure
    M.D. visit

20. What was the reason for your visit?

    Heart problems
    Stroke or transient ischemic attack (TIA), sudden paralysis, vision loss, inability to speak
    Broken, crushed or fractured bones
    Cancer or malignant tumor
    Circulation problem, or blood clots
    Other reasons

21. Please specify the reason for your visit. Hospital Name, Doctor's Name, Location (City, State)

22. Have you stayed overnight as a patient in a nursing home or rehabilitation center since the date of when you downloaded the eFHS app/or completed your medical history update?

23. Please list the name and location of the nursing home or rehabilitation center and the date you were admitted.

24. Nursing home/rehabilitation center name? Location (city, State)

25. Date you entered the nursing home or rehabilitation center

26. Were you an overnight patient in a nursing home or rehabilitation center at any other time since the date of when you downloaded the eFHS app/or completed your medical history update?

27. Please list the name and location of the nursing home or rehabilitation center and the date you were admitted.

28. Nursing home/rehabilitation center name

29. Location (City, State)

30. Date you entered the nursing home or rehabilitation center?

31. Now we are going to ask you some questions about your general status.

32. What is your current marital status? (Select one)

    Single, never married
    Married
    Separated
    Divorced
    Widowed
    Living with partner
    Prefer not to answer

33. Compare your health to most people your own age. Would you say your health is:

    Better
    About the same
    Worse than most people
    Don't know

34. Please list the name and address of your primary care physician. Name? Location (city, State)

- **Health Survey**

1. In general, would you say your health is:

   4 Excellent
   3 Very Good
   2 Good
   1 Fair
   0 Poor

2. During a typical day, does your health now limit you in these activities? If so, how much? Moderate activities, such as moving a table, pushing a vacuum cleaner, bowling, or playing golf

   2 Yes, limited a lot
   1 Yes, limited a little
   0 No, not limited at all

3. During a typical day, does your health now limit you in these activities? If so, how much? Climbing several flights of stairs

   2 Yes, limited a lot
   1 Yes, limited a little
   0 No, not limited at all

4. During the past 4 weeks, have you had any of the following problems with your work or other regular daily activities as a result your physical health?
   Accomplished less than you would like

5. During the past 4 weeks, have you had any of the following problems with your work or other regular daily activities as a result your physical health?

   Were limited in the kind of work or other activites

6. During the past 4 weeks, have you had any of the following problems with your work or other regular daily activities as a result of any emotional problems (such as feeling depressed or anxious)?

   Accomplished less than you would like

7. During the past 4 weeks, have you had any of the following problems with your work or other regular daily activities as a result of any emotional problems (such as feeling depressed or anxious)?
   Didn't do work or other activities as carefully as usual

8. During the past 4 weeks, how much did pain interfere with your normal work (including both work outside the home and housework)?

   0 Not at all
   1 A little bit
   2 Moderately
   3 Quite a bit
   4 Extremely

9. The following questions are about how you feel and how things have been with you during the PAST 4 WEEKS. For each question, please give the one answer that comes closest to the way you have been feeling.

10. How much of the time during the past 4 weeks have you felt calm and peaceful?

    5 All of the time
    4 Most of the time
    3 A good bit of the time
    2 Some of the time
    1 A little of the time
    0 None of the time

11. How much of the time during the past 4 weeks did you have a lot of energy?

    5 All of the time
    4 Most of the time
    3 A good bit of the time
    2 Some of the time
    1 A little of the time
    0 None of the time

12. How much of the time during the past 4 weeks have you felt downhearted and blue?

    5 All of the time
    4 Most of the time
    3 A good bit of the time
    2 Some of the time
    1 A little of the time
    0 None of the time

13. During the past 4 weeks, how much of the time has your physical health or emotional problems interfered with your social activities (like visiting friends, relatives, etc.)?

    4 All of the time
    3 Most of the time
    2 Some of the time
    1 A little of the time
    0 None of the time

Supplementary Table 2: Characteristics of participants in eFHS Participants vs those who did not enroll in the eFHS

Variable	eFHS (n=1948)	Not eFHS (n=1566)	P value*
Age, years, mean (SD)	52.8 (8.7)	56.6 (9.7)	<0.001
Age Groups, n (%)			<0.001
$Age < 45$	330 (16.9)	165 (10.5)	
$45 \leq Age < 55$	782 (40.1)	456 (29.1)	
$55 \leq Age < 65$	670 (34.4)	648 (41.4)	
$Age \geq 65$	166 (8.5)	297 (19.0)	
Female sex, n (%)	1109 (57.0)	782 (49.9)	<0.001
Race (White), n (%)	1813 (93.1)	1414 (90.3)	0.003
Body mass index, kg/m^2 , mean (SD)	28 (6)	29 (6)	<0.001
Systolic blood pressure, mmHg , mean (SD)	119 (14)	121 (15)	<0.001
Diastolic blood pressure, mmHg , mean (SD)	75.84 (8.35)	75.56 (9.00)	0.331
Current smoking, n (%)	113 (5.8)	131 (8.4)	0.004
Diabetes mellitus, n (%)	122 (6.3)	189 (12.4)	<0.001
Hypertension, n (%)	511 (26.3)	582 (37.3)	<0.001
Prevalent cardiovascular disease, n (%)	59 (3.0)	84 (5.4)	<0.001
Physical activity index, mean (SD)	33.6 (5.1)	34.4 (6.2)	<0.001
Highest Education Level Achieved			<0.001
Less than or completed high school	181 (9.3)	336 (21.7)	
Completed some college	467 (24.1)	441 (28.5)	
Bachelor's degree	741 (38.2)	479 (31.0)	
Graduate or professional degree	550 (28.4)	291 (18.8)	
Married, living as married, living with partner, n (%)	1446 (74.7)	1002 (65.1)	<0.001
Subjective Health, n (%)			<0.001
Excellent	468 (24.0)	281 (18.0)	
Very Good	946 (48.6)	632 (40.6)	
Good	451 (23.2)	529 (34.0)	
Poor/Fair	82 (4.2)	116 (7.4)	
Depressive Symptoms Scores, mean (SD)	6 (7)	7 (8)	<0.001
Employed Full Time, n (%)	1361 (70.2)	913 (59.3)	<0.001

* P values reported based on two-sample t-test (for continuous variables) and chi-square test (for nominal/categorical variables)
Data reflect enrollment up to January 28, 2019.
Characteristics were presented as mean ± standard deviation (SD) for continuous variables (for non-skewed distribution), numbers and percentages for nominal variables.

Supplementary Table 3. Definitions of Smartphone App Use, Smartwatch Use, and Digital BP Cuff Use

System Components	N	Deployment Frequency	Definitions of adherence in each eFHS system component
eFHS app	1918	3-months	Completing one survey:<br>Completing 75% of the questions in a given survey<br><br>Definitions of smartphone app use (binary outcome):<br><br>1. Definition 1: Identify participants who complete at least one survey at 3-month window<br>2. Definition 2: Identify participants who complete all surveys at a given survey wave
Smartwatch	1243	Daily	Definition of smartwatch use (binary outcome):<br>Watch wear ≥ 5 hours for at least 1 day per week
BP Cuff	1115	Weekly	Definition of blood pressure (BP) cuff use (binary outcome):<br>Sent BP measurements at least 1 day per week

Smartphone app use =0 at each 3-month period for participants who enrolled in eFHS but never sent any surveys data.
Smartwatch use =0 at each week for participants who took watch but never sent any watch data
Blood pressure cuff use =0 at each week for participants who took blood pressure cuff but never sent any BP data

Supplementary Table 4. Predictor variables selection for final multivariable model of smartphone app use (completing at least one survey)

Predictors	Surveys (univariate) N= 9590 n (eFHSID)= 1918		Surveys (Each individual predictor adjusting for age, sex, and 3-month period) N= 9590 n (eFHSID)=1918	
	OR (95% CI)	P-value*	OR (95% CI)	P-value*
Age				
$Age < 45$	-	-	-	-
$45 \leq Age < 55$	1.03 (0.92-1.16)	0.566		
$55 \leq Age < 65$	1.29 (1.14-1.45)	<0.001		
$Age \geq 65$	1.60 (1.35-1.90)	<0.001		
Sex (Female)	1.29 (1.18-1.39)	<0.001		
Time period				
Baseline	-	-		
3-months	0.17 (0.15-0.20)	<0.001		
6-months	0.14 (0.12-0.16)	<0.001		
9-months	0.09 (0.07-0.10)	<0.001		
12-months	0.08 (0.07-0.10)	<0.001		
Subjective Health				
Excellent	-	-	-	-
VeryGood	0.95 (0.86-1.05)	0.308	0.83 (0.47-1.48)	0.528
Good	0.83 (0.74-0.93)	0.002	0.52 (0.27-1.02)	0.057
Poor/Fair	0.85 (0.69-1.05)	0.138	0.65 (0.19-2.22)	0.487
Depressive Symptoms Scores (scaled)	0.95 (0.91-0.99)	0.014	0.80 (0.64-1.01)	0.058
White	0.90 (0.76-1.05)	0.179	0.67 (0.27-1.67)	0.393
BMI (scaled)	1.00 (0.96-1.04)	0.987	1.04 (0.82-1.32)	0.731
SBP (scaled)	0.99 (0.95-1.03)	0.586	0.90 (0.70-1.16)	0.428
DBP (scaled)	0.96 (0.92-1.00)	0.051	0.99 (0.78-1.26)	0.923

Current Smoking	0.71 (0.60-0.84)	< 0.001	0.32 (0.12-0.82)	0.018
Diabetes	0.95 (0.81-1.13)	0.583	0.80 (0.30-2.12)	0.657
Hypertension	1.00 (0.92-1.10)	0.928	0.79 (0.46-1.37)	0.401
Prevalent of CVD	1.35 (1.06-1.72)	0.015	2.28 (0.56-9.18)	0.248
Physical Activity Index	0.94 (0.90-0.98)	0.002	0.85 (0.67-1.06)	0.153
Highest Education				
Less than or completed high school	-	-	-	-
Completed some college	1.14 (0.98-1.34)	0.094	1.59 (0.66-3.83)	0.307
Bachelor's degree	1.22 (1.05-1.41)	0.009	2.54 (1.09-5.93)	0.030
Graduate or professional degree	1.28 (1.10-1.50)	0.001	3.10 (1.29-7.43)	0.011
Married	1.05 (0.96-1.15)	0.287	1.32 (0.78-2.24)	0.305
Full Time Employment	0.88 (0.80-0.96)	0.004	1.09 (0.64-1.88)	0.745
Cholesterol level (scaled)	0.98 (0.94-1.02)	0.247	0.91 (0.71-1.16)	0.452
Current Alcohol	0.96 (0.87-1.07)	0.461	0.94 (0.52-1.71)	0.852

* P values are derived using Z test statistic from generalized linear mixed models
Outcome is based on Definition 1 of Survey adherence: Proportions of participants who completed at least one survey at baseline and each 3-month window

Supplementary Table 5. Predictor variables selection for final multivariable model of smartphone app use (completing all surveys)

Predictors	Surveys (univariate) N= 9590 n (eFHSID)= 1918		Surveys (Each individual predictor adjusting for age, sex, and weeks) N= 9590 n (eFHSID)=1918	
	OR (95% CI)	P-value*	OR (95% CI)	P-value*
Age				
$Age < 45$	-	-	-	-
$45 \leq Age < 55$	0.97 (0.86-1.09)	0.581		
$55 \leq Age < 65$	1.09 (0.96-1.22)	0.178		
$Age \geq 65$	1.17 (0.98-1.38)	0.077		
Sex (Female)	1.24 (1.14-1.35)	<0.001		
Time period				
Baseline	-	-		
3-months	0.53 (0.46-0.61)	<0.001		
6-months	0.26 (0.22-0.29)	<0.001		
9-months	0.26 (0.23-0.30)	<0.001		
12-months	0.006 (0.001-0.01)	<0.001		
Subjective Health				
Excellent	-	-	-	-
VeryGood	0.95 (0.86-1.05)	0.300	0.87 (0.62-1.22)	0.418
Good	0.85 (0.76-0.96)	0.008	0.68 (0.46-1.01)	0.053
Poor/Fair	0.79 (0.63-0.98)	0.033	0.57 (0.28-1.17)	0.127
Depressive Symptoms Scores (scaled)	0.93 (0.89-0.97	0.001	0.82 (0.71-0.93)	0.003
White	1.05 (0.90-1.24)	0.522	1.11 (0.66-1.88)	0.692
BMI (scaled)	1.01 (0.97-1.05)	0.742	1.05 (0.92-1.21)	0.443
SBP (scaled)	0.98 (0.94-1.02)	0.347	0.97 (0.83-1.12)	0.641
DBP (scaled)	0.98 (0.94-1.02)	0.301	1.03 (0.89-1.18)	0.689
Current Smoking	0.83 (0.69-0.98)	0.032	0.62 (0.35-1.10)	0.105

Diabetes	0.97 (0.82-1.15)	0.716	0.96 (0.54-1.70)	0.892
Hypertension	0.97 (0.89-1.07)	0.538	0.89 (0.65-1.23)	0.494
Prevalent of CVD	1.13 (0.89-1.42)	0.310	1.31 (0.60-2.85)	0.502
Physical Activity Index	0.96 (0.92-1.00)	0.057	0.95 (0.83-1.09)	0.461
Education Level				
Less than or completed high school	-	-	-	-
Completed some college	1.24 (1.06-1.46)	0.008	1.68 (1.00-2.84)	0.051
Bachelor's degree	1.31 (1.13-1.53)	<0.001	2.13 (1.29-3.51)	0.003
Graduate or professional degree	1.41 (1.21-1.66)	<0.001	2.51 (1.50-4.20)	<0.001
Married	1.11 (1.01-1.22)	0.035	1.38 (1.01-1.88)	0.042
Full Time Employment	0.93 (0.85-1.02)	0.115	1.04 (0.76-1.42)	0.816
Cholesterol level (scaled)	0.98 (0.94-1.02)	0.390	0.93 (0.81-1.08)	0.348
Current Alcohol	1.07 (0.97-1.19)	0.176	1.28 (0.90-1.80)	0.165

* P values are derived using Z test statistic from generalized linear mixed models
Outcome is based on Definition 2 of Survey adherence: Proportions of participants who completed all surveys at baseline and each 3-month window

Supplementary Table 6. Stratified analysis with smartphone app use of each 3- month period

Exposure	Time Periods N= 9590 n (eFHSID)=1918									
	Baseline N= 1918		3-month N= 1918		6-month N= 1918		9-month N= 1918		12-month N= 1918	
	OR (95% CI)	P-value*	OR (95% CI)	P-value*	OR (95% CI)	P-value*	OR (95% CI)	P-value*	OR (95% CI)	P-value*
Age (scaled)	1.03 (0.89-1.19)	0.662	1.08 (0.99-1.19)	0.099	1.18 (1.08-1.30)	<0.001	1.30 (1.18-1.43)	<0.001	1.37 (1.24-1.50)	<0.001
Sex (Female)	1.59 (1.19-2.12)	0.002	1.41 (1.17-1.70)	<0.001	1.34 (1.12-1.61)	0.002	1.32 (1.10-1.60)	0.004	1.22 (1.01-1.48)	0.039

* P values are derived using Z test statistic from generalized linear mixed models
Outcome is based on Definition 1 of Survey adherence: Proportions of participants who completed at least one survey at baseline and each 3-month window
All stratified models and interaction model adjusted for age, sex, subjective health, depressive symptoms, education level, and current smoking status

P value $Age \times$ 3-month = 0.518
P value $Age \times$ 6-month = 0.024
P value $Age \times$ 9-month = 0.001
P value $Age \times$ 12-month =0.001
P value $Sex \times$ 3-month = 0.668
P value $Sex \times$ 6-month = 0.942
P value $Sex \times$ 9-month = 0.841
P value $Sex \times$ 12-month = 0.373

Supplementary Table 7. Stratified analysis for app use according to different age groups

Exposure	Age<br>Median age =53<br>N= 9590<br>n (eFHSID)= 1918				P-value* for age Interaction
	Age ≤ 53<br>(N= 5065, n=1013)		Age > 53<br>(N= 4525, 905)		
	OR<br>(95% CI)	P-value*	OR<br>(95% CI)	P-value*	
Sex (Female)	2.65<br>(1.38-5.10)	0.004	2.70<br>(1.36-5.39)	0.005	0.794
Time period					
Baseline	-	-	-	-	
3-months	0.00 (0.00-0.01)	<0.001	0.01 (0.00-0.01)	<0.001	0.582
6-months	0.00 (0.00-0.00)	<0.001	0.00 (0.00-0.01)	<0.001	0.030
9-months	0.00 (0.00-0.00)	<0.001	0.00 (0.00-0.00)	<0.001	0.002
12-months	0.00 (0.00-0.00)	<0.001	0.00 (0.00-0.00)	<0.001	0.001
Subjective Health					
Health Excellent	-	-	-	-	
VeryGood	0.62 (0.28-1.40)	0.251	1.55 (0.66-3.64)	0.315	0.120
Good	0.71 (0.27-1.85)	0.477	0.66 (0.24-1.79)	0.417	0.931
Poor/Fair	0.54 (0.09-3.11)	0.490	2.03 (0.30-13.69)	0.466	0.292
Depressive Symptoms Scores (scaled)	0.86 (0.60-1.23)	0.402	0.85 (0.62-1.18)	0.332	0.898
Current Smoking	0.31 (0.08-1.18)	0.085	0.62 (0.14-2.70)	0.526	0.462
Education					
Less than or completed high school	-	-	-		
Completed some college	2.31 (0.56-9.56)	0.249	1.13 (0.36-3.54)	0.834	0.492
Bachelor’s degree	3.53 (0.92-13.59)	0.066	1.43 (0.46-4.46)	0.535	0.310
Graduate or professional degree	3.38 (0.84-13.69)	0.087	2.34 (0.72-7.57)	0.157	0.692

* P values are derived using Z test statistic from generalized linear mixed models
Outcome is based on Definition 1 of Survey adherence: Proportions of participants who completed at least one survey at baseline and each 3-month window
All stratified models and interaction model adjusted for time of use, sex, subjective health, depressive symptoms, Education Level, and current smoking status

Supplementary Table 8. Predictor variables selection for final multivariable model of smartwatch use

Predictors	Watch Users (univariate)<br>N= 64636<br>n (eFHSID)= 1243		Watch Users (Each individual predictor adjusting for age, sex, and weeks)<br>N= 64636<br>n (eFHSID)= 1243	
	OR (95% CI)	P-value*	OR (95% CI)	P-value*
Age				
$Age < 45$	-	-	-	-
$45 \leq Age < 55$	1.06 (1.01-1.10)	0.015		
$55 \leq Age < 65$	1.24 (1.19-1.30)	<0.001		
$Age \geq 65$	1.60 (1.50-1.71)	<0.001		
Sex (Female)	1.15 (1.11-1.19)	<0.001		
Weeks (scaled)	0.63 (0.62-0.64)	<0.001		
Subjective Health				
Excellent	-	-	-	-
Very Good	0.92 (0.88-0.96)	<0.001	0.62 (0.33-1.16)	0.133
Good	0.68 (0.65-0.71)	<0.001	0.25 (0.12-0.52)	<0.001
Poor/Fair	0.75 (0.69-0.81)	<0.001	0.36 (0.09-1.38)	0.137
Depressive Symptoms Scores (scaled)	0.90 (0.89-0.92)	<0.001	0.70 (0.54-0.90)	0.005
White	0.76 (0.72-0.81)	<0.001	0.51 (0.20-1.27)	0.149
BMI (scaled)	0.91 (0.89-0.92)	<0.001	0.76 (0.59-0.98)	0.034
SBP (scaled)	1.02 (1.01-1.04)	0.003	1.02 (0.77-1.35)	0.900
DBP (scaled)	1.00 (0.98-1.01)	0.640	1.05 (0.80-1.357)	0.738
Current Smoking	0.71 (0.66-0.76)	<0.001	0.32 (0.11-0.98)	0.045

Diabetes	0.99 (0.93-1.06	0.822	0.94 (0.31-2.84)	0.914
Hypertension	1.05 (1.02-1.09)	0.005	0.94 (0.51-1.72)	0.829
Prevalent of CVD	1.30 (1.18-1.43)	<0.001	1.55 (0.35-6.91)	0.568
Physical Activity Index	0.98 (0.96-0.99)	0.003	0.99 (0.77-1.28)	0.934
Highest Education				
Less than or completed high school	-	-	-	-
Completed some college	0.96 (0.90-1.02)	0.199	0.72 (0.26-2.00)	0.531
Bachelor's degree	1.03 (0.97-1.10)	0.289	1.29 (0.49-3.41)	0.605
Graduate or professional degree	1.06 (1.00-1.13)	0.058	1.12 (0.42-3.00)	0.827
Married	1.20 (1.15-1.24)	<0.001	1.57 (0.87-2.82)	0.132
Full Time Employment	0.88 (0.85-0.91)	<0.001	1.04 (0.58-1.89)	0.892
Cholesterol level (scaled)	0.99 (0.97-1.00)	0.144	0.99 (0.77-1.31)	0.992
Current Alcohol	1.04 (0.99-1.08)	0.089	1.38 (0.72-2.63)	0.334

* P values are derived using Z test statistic from generalized linear mixed models

Supplementary Table 9. Predictor variables selection for final multivariable model of digital blood pressure cuff use

Predictors	Blood Pressure Cuff (univariate) N= 57980 n (eFHSID)= 1115		Blood Pressure Cuff (Each individual predictor adjusting for age, sex, and weeks) N= 57980 n (eFHSID)=1115	
	OR (95% CI)	P-value*	OR (95% CI)	P-value*
Age				
$Age < 45$	-	-	-	-
$45 \leq Age < 55$	1.31 (1.24-1.39)	<0.001		
$55 \leq Age < 65$	1.82 (1.72-1.93)	<0.001		
$Age \geq 65$	3.05 (2.83-3.29)	<0.001		
Sex (Female)	1.00 (0.96-1.04)	0.992		
Weeks (scaled)	0.67 (0.66-0.68)	<0.001		
Subjective Health				
Excellent				
Very Good	0.96 (0.92-1.00)	0.049	0.89 (0.63-1.24)	0.483
Good	0.84 (0.80-0.88)	<0.001	0.69 (0.47-1.02)	0.065
Poor/Fair	1.08 (0.98-1.20)	0.121	1.01 (0.45-2.24)	0.989
Depressive Symptoms Scores (scaled)	0.94 (0.93-0.96)	<0.001	0.89 (0.77-1.02)	0.093
White	0.86 (0.81-0.92)	0<0.001	0.73 (0.44-1.24)	0.244
BMI (scaled)	1.01 (0.99-1.03)	0.368	0.98 (0.85-1.13	0.783
SBP (scaled)	1.10 (1.08-1.12)	<0.001	1.01 (0.87-1.18)	0.882
DBP (scaled)	1.00 (0.98-1.01)	0.743	1.01 (0.87-1.17)	0.935
Current Smoking	0.82 (0.75-0.89	< 0.001	0.68 (0.36-1.30)	0.243
Diabetes	1.28 (1.19-1.38)	<0.001	1.24 (0.67-2.28)	0.496
Hypertension	1.21 (1.16-1.26)	<0.001	1.12 (0.81-1.56)	0.482
Prevalent of CVD	1.44 (1.31-1.58)	<0.001	1.63 (0.74-3.56)	0.223

Physical Activity Index	1.00 (0.98-1.01)	0.635	0.94 (0.82-1.09)	0.425
Highest Education				
Less than or completed high school	-	-	-	-
Completed some college	1.01 (0.95-1.09)	0.714	1.32 (0.76-2.29)	0.321
Bachelor's degree	0.82 (0.77-0.88)	<0.001	1.03 (0.61-1.74)	0.917
Graduate or professional degree	1.02 (0.95-1.09)	0.650	1.49 (0.87-2.55)	0.142
Married	0.98 (0.94-1.02)	0.266	1.05 (0.75-1.45)	0.784
Full Time Employment	0.80 (0.77-0.83)	<0.001	0.87 (0.63-1.21)	0.410
Cholesterol level (scaled)	0.95 (0.93-0.96)	<0.001	0.90 (0.78-1.04)	0.169
Current Alcohol	0.86 (0.82-0.90)	<0.001	0.81 (0.57-1.16)	0.251

* P values are derived using Z test statistic from generalized linear mixed models

Supplementary Table 10. Stratified analysis with smartwatch use time (Weeks ≤ 26 and Weeks > 26)

Exposure	Weeks (1-52) N= 64636 n (eFHSID)= 1243				P-value* for weeks Interaction
	Weeks ≤ 26 (N=32318)		Weeks > 26 (N= 32318)		
	OR (95% CI)	P-value*	OR (95% CI)	P-value*	
Age (scaled)	1.17 (0.92-1.50)	0.198	3.03 (1.91-4.81)	<0.001	<0.001
Sex (Female)	1.32 (0.80-2.19)	0.275	2.48 (0.96-6.35)	0.059	0.004

* P values are derived using Z test statistic from generalized linear mixed models
All stratified models and interaction model adjusted for age, sex, subjective health, depressive symptoms, BMI and current smoking status

Supplementary Table 11. Stratified analysis with digital blood pressure cuff use time (Weeks ≤ 26 and Weeks > 26)

Exposure	Weeks (1-52)<br>N= 57980<br>n (eFHSID)= 1115				P-value* for weeks Interaction
	Weeks ≤ 26 (N=28990)		Weeks > 26 (N= 28990)		
	OR (95% CI)	P-value*	OR (95% CI)	P-value*	
Age (scaled)	1.45 (1.29-1.64)	<0.001	2.71 (2.10-3.49)	<0.001	<0.001
Sex (Female)	1.03 (0.80-1.32)	0.824	1.16 (0.69-1.94)	0.578	0.124

* P values are derived using Z test statistic from generalized linear mixed models
All stratified models and interaction model adjusted for age, sex, subjective health, and depressive symptoms

Supplementary Table 12. Stratified analysis for smartwatch use according to different age groups (Age ≤ 53 and Age > 53)

Exposure	Age<br>Median age =53<br>N= 64636<br>n (eFHSID)= 1243				P-value* for age Interaction
	Age ≤ 53<br>(N= 34996, n=673)		Age > 53<br>(N= 29640, 570)		
	OR<br>(95% CI)	P-value*	OR<br>(95% CI)	P-value*	
Sex (Female)	1.67<br>(0.80-3.5)	0.173	1.44<br>(0.69-3.01)	0.338	0.786
Weeks (scaled)	0.19<br>(0.18-0.20)	<0.001	0.31<br>(0.30-0.33)	<0.001	<0.001
Subjective Health					
Excellent	-	-	-	-	
Very Good	0.70 (0.28-1.76)	0.451	0.86 (0.34-2.16)	0.745	0.663
Good	0.66 (0.21-2.05)	0.469	0.21 (0.07-0.63)	0.005	0.147
Poor/Fair	0.55 (0.07-3.97)	0.550	1.12 (0.13-9.40)	0.918	0.498
Depressive Symptoms Scores (scaled)	0.73 (0.49-1.10)	0.131	0.76 (0.54-1.06)	0.107	0.896
BMI (scaled)	0.77 (0.51-1.15)	0.205	0.93 (0.64-1.36)	0.716	0.778
Current smoking Status	0.35 (0.07-1.66)	0.185	0.43 (0.09-2.10)	0.296	0.999

* P values are derived using Z test statistic from generalized linear mixed models
All stratified models and interaction model adjusted for time of use, sex, subjective health, depressive symptoms, BMI and current smoking status

Supplementary Table 13. Stratified analysis for digital blood pressure cuff use according to different age groups (Age ≤ 53 and Age > 53)

Exposure	Age<br>Median age =53<br>N= 57980<br>n (eFHSID)= 1115				P-value* for age Interaction
	Age ≤ 53<br>(N=30316, n=583)		Age > 53<br>(N=27664, 532)		
	OR<br>(95% CI)	P-value*	OR<br>(95% CI)	P-value*	
Sex (Female)	1.23<br>(0.85-1.79)	0.278	0.87<br>(0.57-1.32)	0.507	0.212
Weeks (scaled)	0.44<br>(0.43-0.46)	<0.001	0.56<br>(0.54-0.58)	<0.001	<0.001
Subjective Health					
Excellent	-	-	-	-	
Very Good	0.54 (0.34-0.84)	0.007	1.75 (1.06-2.91)	0.030	0.001
Good	0.70 (0.41-1.20)	0.194	0.83 (0.46-1.51)	0.549	0.615
Poor/Fair	0.72 (0.25-2.09)	0.547	2.12 (0.58-7.75)	0.254	0.163
Depressive Symptoms Scores (scaled)	0.88 (0.70-1.09)	0.236	0.89 (0.73-1.09)	0.266	0.723

* P values are derived using Z test statistic from generalized linear mixed models
All stratified models and interaction model adjusted for time of use, sex, subjective health, and depressive symptoms

Supplementary Table 14. Characteristics of eFHS Participants stratified according different age groups

Variable	$Age < 45$ (n=330)	$45 \leq Age < 55$ (n=782)	$55 \leq Age < 65$ (n=670)	$Age \geq 65$ (n=166)	P value*
Female sex, n (%)	193 (58.5)	437 (55.9)	388 (57.9)	91 (54.8)	0.744
Race (White), n (%)	291 (88.2)	732 (93.6)	644 (96.1)	146 (88)	<0.001
Body mass index, kg/m^2, mean (SD)	27.4 (5.7)	28.2 (5.6)	28.5 (5.7)	29.0 (5.6)	0.006
Systolic blood pressure, mmHg , mean (SD)	113 (12)	116 (13)	122 (14)	127 (14)	<0.001
Diastolic blood pressure, mmHg , mean (SD)	75 (8)	76 (9)	76 (8.2)	75 (8)	0.001
Current smoking, n (%)	21 (6.4)	49 (6.3)	39 (5.8)	4 (2.4)	0.261
Diabetes mellitus, n (%)	6 (1.8)	34 (4.4)	58 (8.7)	24 (14.7)	<0.001
Hypertension, n (%)	29 (8.8)	161 (20.6)	229 (34.3)	92 (55.4)	<0.001
Prevalent cardiovascular disease, n (%)	3 (0.9)	13 (1.7)	25 (3.7)	18 (10.8)	<0.001
Physical activity index, mean (SD)	33.76 (4.7)	33.4 (5.2)	33.8 (5.3)	32.9 (4.4)	0.102
Education Level Achieved					<0.001
Less than or completed high school	23 (7.0)	56 (7.2)	82 (12.3)	20 (12.1)	
Completed some college	49 (14.8)	173 (22.2)	195 (29.4)	50 (30.3)	
Bachelor's degree	144(43.6)	336 (43.1)	215 (32.4)	46 (27.9)	
Graduate or professional degree	114 (34.5)	215 (27.6)	172 (25.9)	49 (29.7)	
Married, living as married, living with partner, n (%)	247 (75.1)	593 (76.4)	488 (73.5)	118 (71.1)	0.408
Subjective Health, n (%)					0.878
Excellent	83 (25.2)	181 (23.2)	170 (25.4)	34 (20.5)	
Very Good	155 (47.0)	385 (49.3)	318 (47.5)	88 (53.0)	
Good	77 (23.3)	180 (23.0)	154 (23.0)	40 (24.1)	
Poor/ Fair	15 (4.5)	35 (4.6)	28 (4.2)	4 (2.4)	
Depressive Symptoms Scores, mean (SD)	6 (6)	6 (6)	6 (7)	6 (6)	0.999
Employed Full Time, n (%)	257 (78.1)	614 (78.9)	442 (66.3)	48 (29.1)	<0.001

Data reflect enrollment up to January 28, 2019.
Characteristics were presented as mean ± standard deviation (SD) for continuous variables (for non-skewed distribution), numbers and percentages for nominal variables.
* P values reported based on Analysis of variance / ANOVA (for continuous variables) and chi-square test (for nominal/categorical variables)

Supplementary Table 15. Characteristics of eFHS Participants stratified according type of phone

Variable	Android (n=277)	iPhone (n=1663)	P value
Age, years, mean (SD)	53.1 (8.8)	52.8 (8.7)	0.534
Age Groups, n (%)			0.946
$Age < 45$	46 (16.6)	284 (17.1)	
$45 \leq Age < 55$	108 (39.0)	671 (40.3)	
$55 \leq Age < 65$	98 (35.4)	570 (34.3)	
$Age \geq 65$	25 (9.0)	138 (8.3)	
Female sex, n (%)	131 (47.3)	971 (58.4)	<0.001
Race (White), n (%)	263 (94.9)	1543 (92.8)	0.236
Body mass index, kg/m^2 , mean (SD)	29.1 (5.9)	28.1 (5.6)	<0.001
Systolic blood pressure, mmHg, mean (SD)	121 (14)	118 (14)	<0.001
Diastolic blood pressure, mmHg , mean (SD)	77 (9)	76 (8)	0.029
Current smoking, n (%)	26 (9.4)	87 (5.2)	0.010
Diabetes mellitus, n (%)	22 (8.0)	100 (6.0)	0.275
Hypertension, n (%)	87 (31.5)	420 (25.3)	0.035
Prevalent cardiovascular disease, n (%)	10 (3.6)	49 (2.9)	0.684
Physical activity index, mean (SD)	34.6 (6.1)	33.4 (4.9)	<0.001
Education Level Achieved			<0.001
Less than or completed high school	33 (12.0)	148 (8.9)	
Completed some college	95 (34.4)	371 (22.4)	
Bachelor's degree	88 (31.9)	651 (39.3)	
Graduate or professional degree	60 (21.7)	486 (29.3)	
Married, living as married, living with partner, n (%)	190 (68.8)	1250 (75.7)	0.018
Subjective Health, n (%)			0.020
Excellent	49 (17.7)	418 (25.2)	
Very Good	147 (53.1)	795 (47.8)	
Good	64 (23.1)	385 (23.2)	
Poor/ Fair	17 (6.1)	64 (3.9)	
Depressive Symptoms Scores , mean (SD)	6 (7)	6 (7)	0.759
Employed Full Time, n (%)	198 (71.5)	1161 (70.2)	0.717

* P values reported based on two-sample t-test (for continuous variables) and chi-square test (for nominal/categorical variables)
Data reflect enrollment up to January 28, 2019.
Characteristics were presented as mean ± standard deviation (SD) for continuous variables (for non-skewed distribution), numbers and percentages for nominal variables.
