# Supplementary material for: Factors associated with long-term use of digital devices in the electronic Framingham Heart Study
Source: NPJ Digit Med. 2022 Dec 27;5:195. doi: 10.1038/s41746-022-00735-1 (PMC9792462; doi:10.1038/s41746-022-00735-1)
Supplement: Supplementary file 2 — Reporting Summary [file 41746_2022_735_MOESM2_ESM.pdf]

nature portfolio

Corresponding author(s): Chathurangi H. Pathiravasan

Last updated by author(s): 9/16/2022

Reporting Summary

Nature Portfolio wishes to improve the reproducibility of the work that we publish. This form provides structure for consistency and transparency in reporting. For further information on Nature Portfolio policies, see our Editorial Policies and the Editorial Policy Checklist.

Statistics

For all statistical analyses, confirm that the following items are present in the figure legend, table legend, main text, or Methods section.

n/a	Confirmed	
☐	☒	The exact sample size (*n*) for each experimental group/condition, given as a discrete number and unit of measurement
☐	☒	A statement on whether measurements were taken from distinct samples or whether the same sample was measured repeatedly
☐	☒	The statistical test(s) used AND whether they are one- or two-sided<br>*Only common tests should be described solely by name; describe more complex techniques in the Methods section.*
☐	☒	A description of all covariates tested
☐	☒	A description of any assumptions or corrections, such as tests of normality and adjustment for multiple comparisons
☐	☒	A full description of the statistical parameters including central tendency (e.g. means) or other basic estimates (e.g. regression coefficient) AND variation (e.g. standard deviation) or associated estimates of uncertainty (e.g. confidence intervals)
☐	☒	For null hypothesis testing, the test statistic (e.g. *F*, *t*, *r*) with confidence intervals, effect sizes, degrees of freedom and *P* value noted<br>*Give P values as exact values whenever suitable.*
☒	☐	For Bayesian analysis, information on the choice of priors and Markov chain Monte Carlo settings
☐	☒	For hierarchical and complex designs, identification of the appropriate level for tests and full reporting of outcomes
☐	☒	Estimates of effect sizes (e.g. Cohen's *d*, Pearson's *r*), indicating how they were calculated

Our web collection on statistics for biologists contains articles on many of the points above.

Software and code

Policy information about availability of computer code

Data collection	In person's visits of FHS participants, data collected through smartwatch, BP Cuff, smartwatch app.<br>All statistical analyses were performed with R software (R version 4.0.5) and the lme4 package in R was used for analysis with GLMMs.
Data analysis	Code for the analysis is available upon reasonable request from the corresponding author.

For manuscripts utilizing custom algorithms or software that are central to the research but not yet described in published literature, software must be made available to editors and reviewers. We strongly encourage code deposition in a community repository (e.g. GitHub). See the Nature Portfolio guidelines for submitting code & software for further information.

Data

Policy information about availability of data

All manuscripts must include a data availability statement. This statement should provide the following information, where applicable:

- Accession codes, unique identifiers, or web links for publicly available datasets
- A description of any restrictions on data availability
- For clinical datasets or third party data, please ensure that the statement adheres to our policy

Data of the eFHS will be available at the Biologic Specimen and Data Repository Information Coordinating Center (BioLINCC). BioLINCC has a two-year release policy but data will be accessible to investigators via the authors until released by BioLINCC.

Human research participants

Policy information about studies involving human research participants and Sex and Gender in Research.

Reporting on sex and gender	Here we are considering sex (biological attribute) not the gender and findings are applicable only for sex groups. Sex was determined based on self-reporting. Consent has been obtained for sharing individual-level data. We provided overall numbers in this reporting summary. We provided sex based analysis.
Population characteristics	Sociodemographic: self-reported age, sex, marital status, race/ethnicity, education, and employment status.<br><br>Lifestyle: smoking, alcohol, and physical activity<br><br>CVD risk factors and medical conditions: body mass index (BMI), cholesterol (mg/dL), systolic blood pressure (SBP, mmHg), diastolic blood pressure (DBP, mmHg), hypertension, diabetes mellitus, and prevalent CVD (defined below). Cholesterol levels
Recruitment	Participants were recruited from the FHS Third Generation (Gen 3) cohort (n= 4095), multiethnic Omni Group 2 Cohort (n=410) and New Offspring Spouse (n=103) who were initially enrolled into the FHS from 2002 to 2005. To be eligible, eFHS participants attended exam 3 (2016-2019), owned a smartphone (Android or compatible iPhone iOS version 9 or higher). All participants provided written informed consent (two steps: first step at exam 3 and electronic consent as the second step within the eFHS app) and written eFHS protocol. eFHS participants owned a smartphone, primarily resided in the New England region of the United States and they were more likely to be White, well educated, and reported excellent health. Thus, the findings may not be generalizable to more diverse samples.
Ethics oversight	The eFHS study was reviewed and approved by the Institutional Review Board at Boston University Medical Center.

Note that full information on the approval of the study protocol must also be provided in the manuscript.

Field-specific reporting

Please select the one below that is the best fit for your research. If you are not sure, read the appropriate sections before making your selection.

☒ Life sciences ☐ Behavioural & social sciences ☐ Ecological, evolutionary & environmental sciences

For a reference copy of the document with all sections, see nature.com/documents/nr-reporting-summary-flat.pdf

Life sciences study design

All studies must disclose on these points even when the disclosure is negative.

Sample size	A modestly sized (n≈1948) community-based sample of middle-aged adults unselected for any health condition (Observational study)
Data exclusions	Among 2,151 participants enrolled in the eFHS, 203 individuals (who enrolled from Feb 2019- Aug 2019) were excluded due to being followed for less than 12-months from enrollment. When we started the analysis this information was not available.
Replication	N/A
Randomization	N/A
Blinding	N/A

Reporting for specific materials, systems and methods

We require information from authors about some types of materials, experimental systems and methods used in many studies. Here, indicate whether each material, system or method listed is relevant to your study. If you are not sure if a list item applies to your research, read the appropriate section before selecting a response.

Materials & experimental systems

n/a	Involved in the study
☒	☐ Antibodies
☒	☐ Eukaryotic cell lines
☒	☐ Palaeontology and archaeology
☒	☐ Animals and other organisms
☐	☒ Clinical data
☒	☐ Dual use research of concern

Methods

n/a	Involved in the study
☒	☐ ChIP-seq
☒	☐ Flow cytometry
☒	☐ MRI-based neuroimaging

Clinical data

Policy information about clinical studies

All manuscripts should comply with the ICMJE guidelines for publication of clinical research and a completed CONSORT checklist must be included with all submissions.

Clinical trial registration	*Provide the trial registration number from ClinicalTrials.gov or an equivalent agency.*
Study protocol	*Note where the full trial protocol can be accessed OR if not available, explain why.*
Data collection	*Describe the settings and locales of data collection, noting the time periods of recruitment and data collection.*
Outcomes	*Describe how you pre-defined primary and secondary outcome measures and how you assessed these measures.*
